# Reductive Elimination from Sterically Encumbered Ni–Polypyridine Complexes

**DOI:** 10.1021/acs.organomet.2c00362

**Published:** 2022-09-21

**Authors:** Craig S. Day, Stephanie J. Ton, Ryan T. McGuire, Cina Foroutan-Nejad, Ruben Martin

**Affiliations:** † The Barcelona Institute of Science and Technology, Institute of Chemical Research of Catalonia (ICIQ), Av. Països Catalans 16, 43007 Tarragona, Spain; ‡ Departament de Química Analítica i Química Orgànica, Universitat Rovira i Virgili, c/Marcel·lí Domingo 1, 43007 Tarragona, Spain; § Institute of Organic Chemistry, Polish Academy of Sciences, Kasprzaka 44/52, 01-224 Warsaw, Poland; ∥ ICREA, Passeig Lluís Companys 23, 08010 Barcelona, Spain

## Abstract

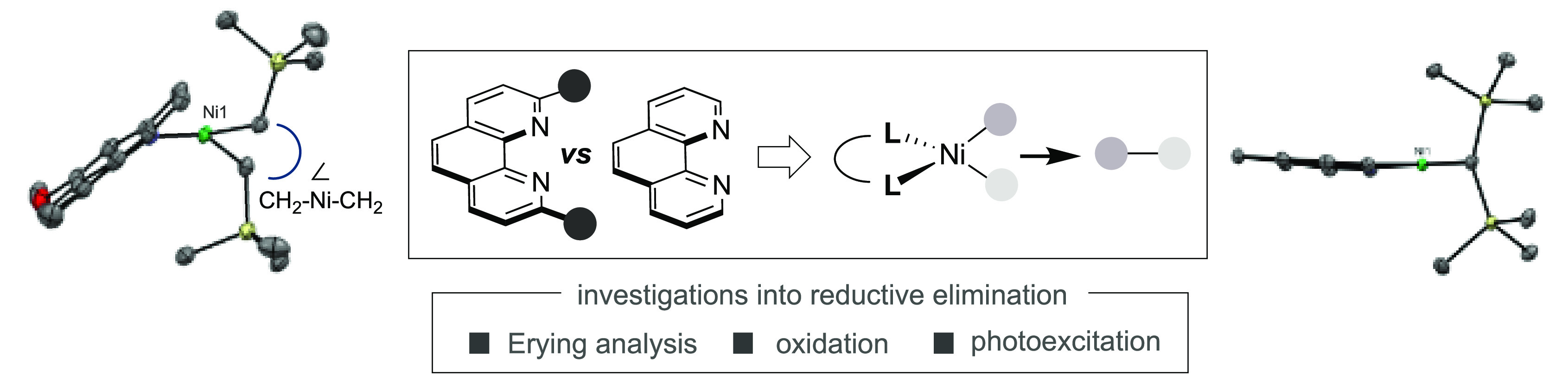

Herein we disclose the synthesis of sterically encumbered dialkylnickel(II) complexes bearing 2,9-dimethyl-1,10-phenanthroline ligands. A comparison with their unsubstituted analogues by both X-ray crystallography and theoretical calculations revealed significant distortions in their molecular structures. Eyring plots along with stoichiometric and photoexcitation studies revealed that sterically encumbered dialkylnickel(II) complexes enable facile C(*sp*^3^)–C(*sp*^3^) reductive elimination, thus offering an improved understanding of Ni catalysis.

Nickel-catalyzed reactions have gained considerable momentum as enabling techniques for forging new synthetic architectures.^1−4^ Particularly attractive is the virtue of Ni catalysts for forging C(*sp*^3^)–C(*sp*^3^) bonds, as these bonds are key motifs in medicinal chemistry programs that modulate solubility, molecular shape, or substrate recognition of drug candidates.^5−8^ The successful implementation of nickel catalysis in both academic and industrial laboratories is intimately associated with the ease of enabling single-electron-transfer reactivity, the propensity to populate unconventional Ni(I) or Ni(III) manifolds, and the high barrier for β-hydride elimination that allows for forging of *sp*^3^ architectures.^9^ Reviewing the literature data reveals that sterically encumbered polypyridine ligands have proved to be a key contributory factor for success in a myriad of Ni-catalyzed C(*sp*^3^)–C(*sp*^3^) bond formations.^10^ Although there exists a reasonable consensus on how Ni–polypyridine complexes enable oxidative addition or transmetalation, the means to trigger C(*sp*^3^)–C(*sp*^3^) reductive elimination remains the subject of considerable debate due to the inherent difficulty of accessing short-lived yet exceptionally sensitive dialkylnickel(II)–polypyridine species (Scheme 1, top).^11−18^ Seminal studies by Yamamoto et al. determined that the barrier to reductive elimination from (bpy)NiEt_2_ was 68 kcal·mol^–1^.^19−24^ The extensive literature on the steric implications of phosphine ligands for reductive elimination and the rapid adoption of sterically encumbered polypyridine ligands for the construction of C(*sp*^3^)–C(*sp*^3^) bonds prompted our interest in studying these systems further (Scheme 1, middle).^25,26^ We anticipated that a study aimed at unraveling the steric effects of dialkylnickel–polypyridine complexes on C(*sp*^3^)–C(*sp*^3^) reductive elimination might represent a new lead for future Ni-catalyzed cross-coupling reactions (Scheme 1, bottom).

**Scheme 1:**
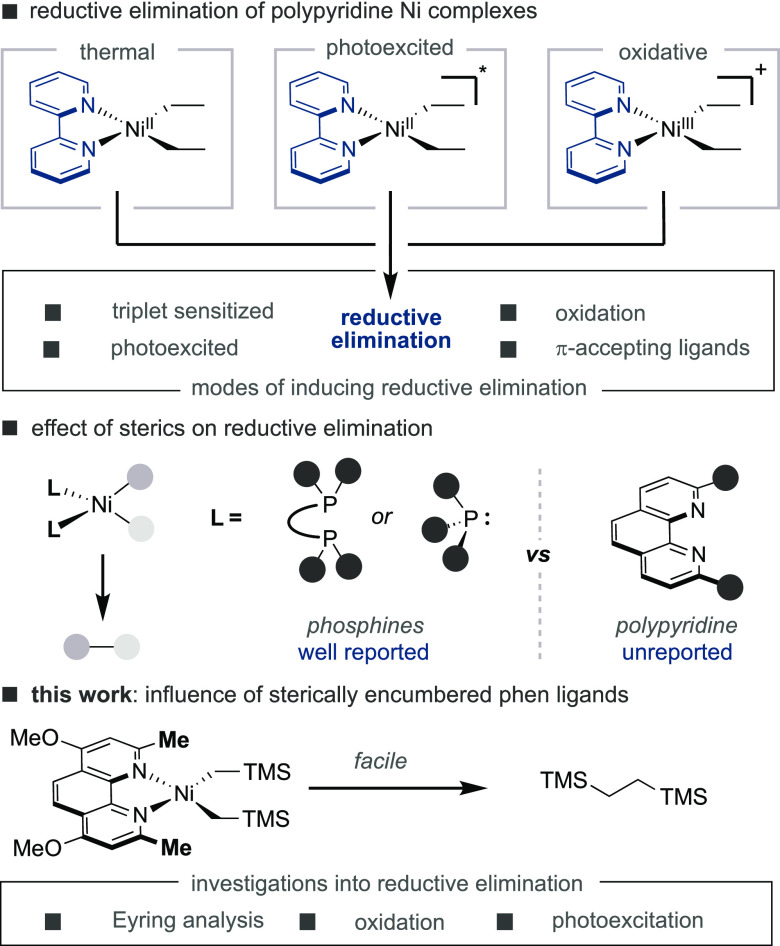
Reductive Elimination of Polypyridine-Ligated Ni Complexes

We began our investigations by synthesizing well-defined dialkylnickel(II) complexes bearing variously substituted polypyridine ligands.^21^ Specifically, we allowed Ni(acac)_2_ to react with Et_2_AlOEt in the presence of either 2,2′-bipyridine (**L1**) or neocuproine (**L2**) in Et_2_O at −20 °C (Figure 1). While the synthesis of (**L1**)NiEt_2_ (**1**) posed no problems, the preparation of (**L2**)NiEt_2_ was found to be particularly problematic, as (**L2**)Ni(ethylene) crystallized from the reaction mixture in 60% yield. This product, corroborated by X-ray diffraction, presumably arises from β-hydride elimination. The exceptional ease with which (**L2**)NiEt_2_ undergoes β-hydride elimination is in sharp contrast with the high barrier observed in the analogous reaction of (**L1**)NiEt_2_. The importance of this observation can hardly be overestimated, as it offers indirect yet long-awaited evidence for the propensity of 2,9-disubstituted phenanthroline to enable Ni-catalyzed chain walking via iterative β-hydride elimination and migratory insertion events.^27^

**Figure 1:**
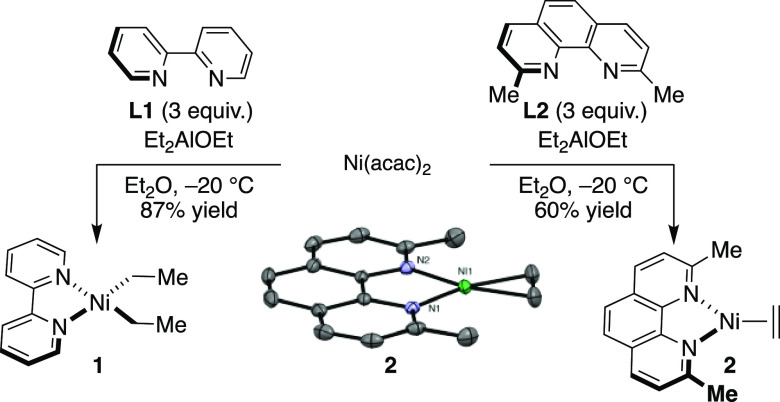
Initial efforts en route to dialkylnickel(II) species.

Aiming to understand the factors influencing reductive elimination of Ni(dialkyl) complexes supported by sterically encumbered polypyridine ligands and the difficulties of synthesizing L_2_NiR_2_ complexes by the use of alkylaluminum reagents, we sought out a pathway involving neutral ligand displacement. To this end, we turned our attention to (py)_2_Ni(CH_2_TMS)_2_ (TMS = trimethylsilyl), which is easily prepared by reaction of (py)_4_NiCl_2_ with TMSCH_2_MgCl in Et_2_O at −60 °C.^28^ This nickel precursor was chosen because of the ease with which monodentate pyridine ligands could be displaced by 1,10-phenanthroline ligands. Furthermore, we anticipated that the CH_2_TMS groups would add to the stability of the complexes by hyperconjugation and by preventing β-hydride elimination.^29−32^ As expected from early studies reported by Carmona and Atwood, (**L3**)Ni(CH_2_TMS)_2_ (**3**) was prepared in 90% yield by the reaction between (py)_2_Ni(CH_2_TMS)_2_ and 1,10-phenanthroline (**L3**) at room temperature.^28^ Notably, the reaction between (py)_2_Ni(CH_2_TMS)_2_ and 4,7-dimethoxy-2,9-dimethyl-1,10-phenanthroline (**L4**)—a ligand featuring electron-donating methoxy groups via resonance donation to the ligand π system—could be conducted at −36 °C, giving rise to (**L4**)Ni(CH_2_TMS)_2_ (**4**) in 68% yield as a purple solid (Figure 2). The stability of this complex may arise from the electron-richness of the metal center, which prevents reductive elimination. In keeping with this hypothesis, not even traces of (**L5**)Ni(CH_2_TMS)_2_ (**L5** = 2,9-dimethyl-4,7-diphenyl-1,10-phenanthroline) were observed upon the reaction of (py)_2_Ni(CH_2_TMS)_2_ with **L5**, a ligand with inductively withdrawing phenyl groups in place of the methoxy groups of **L4**. The preparation of **4** is particularly important, as it offers for the first time the opportunity to assess the influence of sterically encumbered polypyridine complexes in the context of C(*sp*^3^)–C(*sp*^3^) reductive elimination from a well-defined species. This information may therefore allow the parametrization of features that may have an impact in future Ni-catalyzed endeavors.

**Figure 2:**
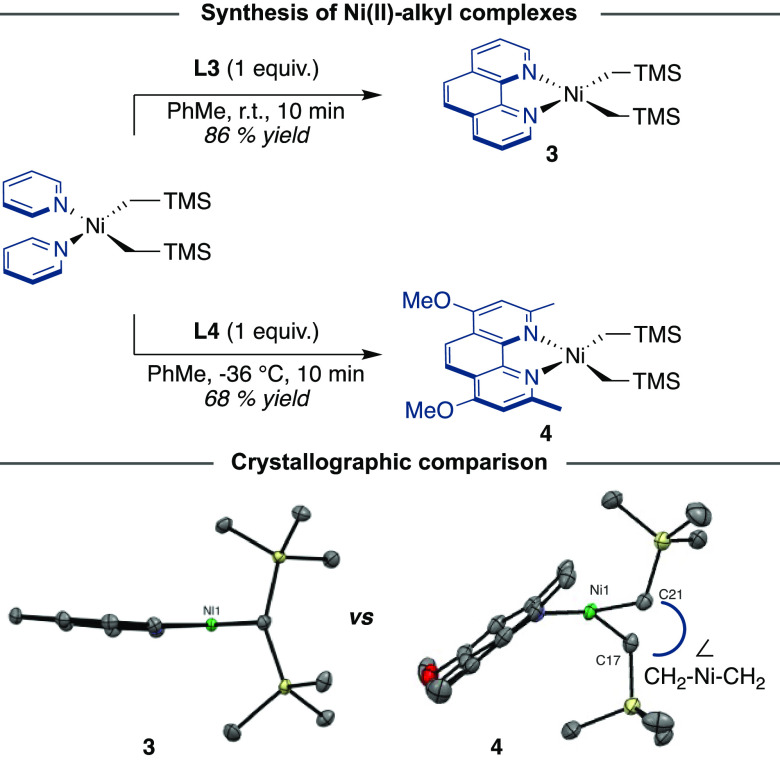
Synthesis of (**L**)Ni(CH_2_TMS)_2_ (**L** = **L3**, **L4**) and ORTEP drawings (50%) of **3** and **4**. Crystals of **3** and **4** were grown at −36 °C in Et_2_O/pentane or Et_2_O. Hydrogen atoms and disordered sections have been omitted in the sake of clarity.

Low-temperature crystallization (−36 °C, Et_2_O/pentane or Et_2_O) furnished crystals suitable for X-ray diffraction, thus allowing the structures of **3** and **4** to be determined unambiguously. A simple comparison of their structures is particularly illustrative. While the Ni atom in **3** is in a canonical square planar geometry, the bonding in **4** is fairly distorted from a square planar geometry, although the complex is still diamagnetic. DFT calculations of the gas-phase structure of **4** confirmed that this nonplanarity is a molecular phenomenon, not a result of the crystal packing. Strikingly, the coordination of **L4** is ligated at a ca. 35° angle, which results in poor overlap of the σ-sp^2^ orbital of nitrogen with the central Ni atom. This is reflected in lower values of the delocalization index δ(A, B)—a measure of the orbital overlap and covalent bond order between a pair of atoms A and B—computed within the context of the quantum theory of atoms in molecules (QTAIM):^33^ in **4**, δ(Ni, N) = 0.459 and 0.394, whereas in **3**, δ(Ni, N) = 0.499. Natural bond orbital analysis further confirmed a weaker overlap between the lone pairs of the nitrogen atoms and the p orbital of Ni in **4**. While second-order perturbation theory for the symmetrical structure of **3** predicted two equally strong interactions from the nitrogen atoms of **L3** (*E*(2) = 35.2 kcal·mol^–1^), a significant deviation is observed in nonsymmetrical **L4** (*E*(2) = 33.6 and 33.9 kcal·mol^–1^). We speculate that this indirect binding mode might limit steric pressure surrounding the Ni center, as a direct binding interaction might locate the 2,9-dimethyl substituents of **L4** in close proximity to the methylene carbons on the CH_2_TMS moiety.

Further inspection of the X-ray structure of **4** identified a heavily distorted square planar geometry, with the methylene carbon C17 significantly out of the N1–Ni–N2 plane. A seemingly simple comparison of the CH_2_–Ni–CH_2_ angle from the side as shown in Figure 2 reveals that the methylene carbons are distorted by 38.9°, which is in contrast to the distortion of 2.8° observed for **3**. Further comparison of the two structures revealed that **4** has longer N–Ni bonds (2.055(3) and 1.982(3) Å in **4** vs 1.9833(16) and 1.9886(15) in **3**) and contracted Ni–C linkages (1.930(3) and 1.933(3) Å in **4** vs 1.9441(18) and 1.9442(18) Å in **3**). Comparing the QTAIM atomic charges (*q*(Ni)) and localization index (λ(Ni)) of **3** and **4** suggests that **L4** in **4** increases the electron population of Ni more than does coordination of **L3** in **3** (*q*(Ni)_**4**_ = +0.612 and λ(Ni)_**4**_ = 25.755 vs *q*(Ni)_**3**_ = +0.635 and λ(Ni)_**3**_ = 25.699). Analysis of the frontier molecular orbitals of **3** and **4** reveals that the complexation with **L4** increases the HOMO energies of **4** more than those of **3** (Figure 3), which is consistent with the highly strained geometry of **4**. Consistent with **4** containing a higher-energy HOMO were cyclic voltammetry experiments which found that Ni(II/III) oxidation is easier in **4** (*E*_ox_ = −0.40 V vs SCE) than in **3** (*E*_ox_ = 0.22 V vs SCE).

**Figure 3:**
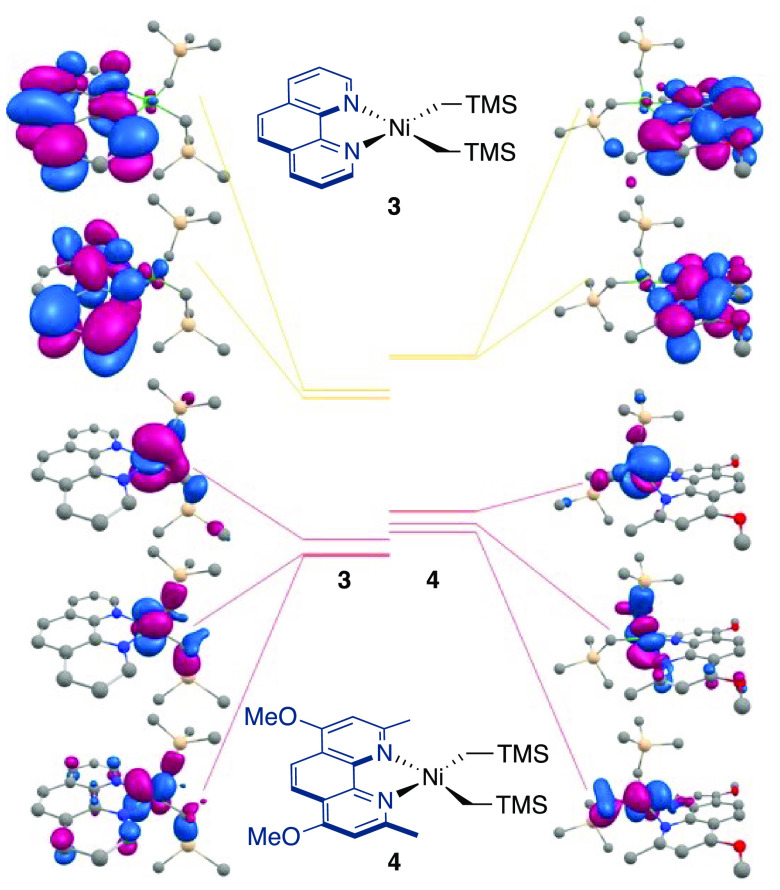
Frontier molecular orbitals of (left) **3** and (right) **4** by DFT.

In order to study the ligand effects of **L3** and **L4** on C–C reductive elimination from **3** and **4**, respectively, we monitored these compounds at elevated temperature in C_6_D_6_. Demonstrating the stability of **3**, even after 24 h at 100 °C, **3** showed no reaction. The striking stability of **3** is in sharp contrast to its sterically encumbered analogue **4**, which underwent reductive elimination in C_6_D_6_ at 60 °C with a first-order decay (*k* = 3.72 × 10^–4^ s^–1^) over 100 min to form insoluble (**L4**)_2_Ni and 1,2-bis(trimethylsilyl)ethane (Figure 4).

**Figure 4:**
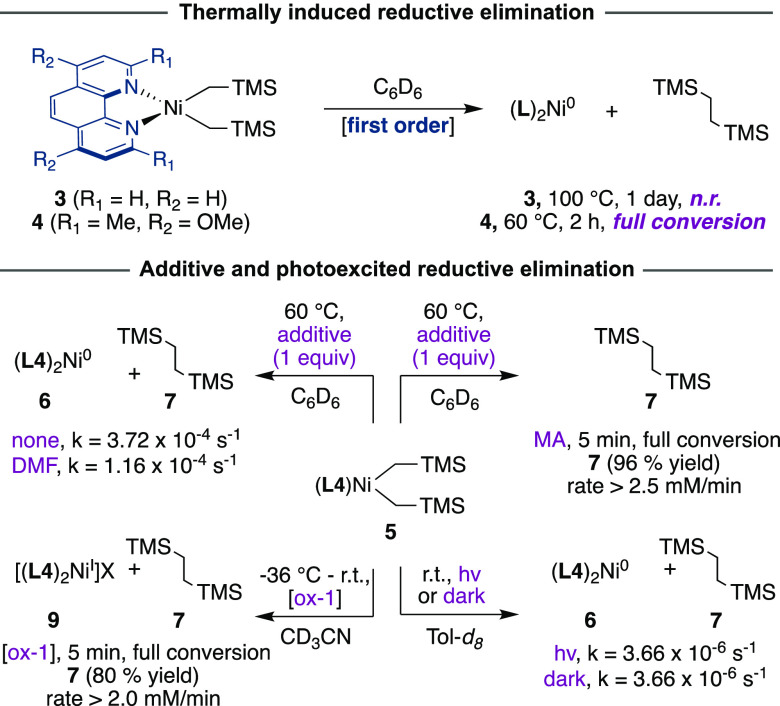
Reductive elimination of TMSCH_2_CH_2_TMS from **4** under various conditions: thermally, with additives, under ambient light and with an oxidant. ox-1 = 1-fluoro-2,4,6-trimethylpyridinium tetrafluoroborate.

Further information on the reductive elimination was gathered by performing an Eyring analysis in C_6_D_6_, which determined a particularly low activation barrier (Δ*G*^⧧^(50 °C) = 26.3 kcal·mol^–1^). Qualitative data on the transition state could be obtained by performing a preliminary three-point Eyring plot analysis in THF-*d*_8_ (50–70 °C) with single kinetic runs, from which a negative entropy of activation is apparent (Figure S11).^34^ To gain additional information on the effect of coordinating ligands, we examined whether the inclusion of methyl acrylate (MA) might influence reductive elimination in **4**. In line with studies performed by Yamamoto with (bpy)NiEt_2_, the presence of the π-accepting olefin MA induced rapid reductive elimination (<5 min), which we speculate is due to the intermediacy of five-coordinate species via interaction with the d_*x*^2^–*y*^2^_ orbital (Figure 4).^35^ Taking into consideration that metallaphotoredox scenarios have gained considerable momentum as innovative vehicles for forging *sp*^3^ architectures, we next focused our attention on studying whether C(*sp*^3^)–C(*sp*^3^) reductive elimination of **4** might be facilitated by either photoexcitation or single-electron-transfer oxidation. Notably, a rate enhancement similar to that observed for MA was observed when the reaction of **4** was conducted with 1-fluoro-2,4,6-trimethylpyridinium tetrafluoroborate, which acts as a one-electron oxidant,^36^ thus illustrating the exceptional ease with which Ni(III) intermediates promote reductive elimination. Comparing the rate of reductive elimination of **4** in the dark or under ambient light (**4** absorbs (λ_max_ = 399 nm)) revealed a similar rate of reaction (*k* = 3.66 × 10^–6^s^–1^).^37^ These observations were further corroborated by the lack of changes in the crystal structure of **4** when crystals of **4** were irradiated at 390 nm.

In summary, we have reported the first dialkylnickel(II) complex supported by sterically encumbered 2,9-disubstituted phenanthroline ligands. A comparison of the solid-state geometry with that of its unsubstituted analogue reveals that steric effects destabilize these complexes, setting the basis for promoting C(*sp*^3^)−C(*sp*^3^) reductive elimination with exceptional ease. Stoichiometric experiments were carried out in different solvents, in the presence of additives, and in the presence of light. We believe that this report might lead to new knowledge in synthetic design while offering a new gateway for studying the intricacies of Ni-catalyzed reactions.

## Supporting Information Available

Cartesian coordinates for **3** (XYZ)
Cartesian coordinates for **4** (XYZ)
Experimental procedures and spectral and crystallographic data (PDF)

## Supplementary Material

om2c00362_si_001.xyz

om2c00362_si_002.xyz
