# Supplementary material for: Reductive Elimination from Sterically Encumbered Ni–Polypyridine Complexes
Source: Organometallics. 2022 Sep 21;41(19):2662–7. doi: 10.1021/acs.organomet.2c00362 (PMC9554914; doi:10.1021/acs.organomet.2c00362)
Supplement: Supplementary file 3 — om2c00362_si_003.pdf [file om2c00362_si_003.pdf]

Go to: [table of contents] [references]

Supporting Information

Reductive elimination from sterically encumbered Ni-polypyridine complexes

Craig S. Day,[†£] Stephanie J. Ton,[†#] Ryan T McGuire,[†#] Cina Foroutan-Nejad,[*$] and Ruben Martin[†§*]

[†] Institute of Chemical Research of Catalonia (ICIQ), The Barcelona Institute of Science and Technology, Av. Països Catalans 16, 43007 Tarragona, Spain

[£] Departament de Química Analítica i Química Orgànica, Universitat Rovira i Virgili, c/Marcel·lí Domingo, 1, 43007 Tarragona, Spain

[$] Institute of Organic Chemistry, Polish Academy of Sciences, Kasprzaka 44/52, 01-224, Warsaw, Poland.

[§] ICREA, Passeig Lluís Companys, 23, 08010 Barcelona, Spain

[#] S. J. T., and R. T. M. contributed equally to this work

Corresponding authors: rmartinromo@iciq.es, cforoutan-nejad@icho.edu.pl

Table of Contents

S1. General Considerations 2
S2. Synthesis and Characterization of Complexes 3
  Synthesis of (L4)Ni(CH_2TMS)$_2$ 3
S3. Stochiometric Reactions. 4
  Instability of (Bc)Ni(CH_2TMS)$_2$ and formation of (Bc)$_2$Ni 4
  Attempting analogous synthesis to (bipy)NiEt$_2$ with neocuproine 5
  Reductive elimination of (Phen)Ni(CH_2TMS)$_2$ 6
  Independent synthesis of (L4)$_2$Ni 7
  Monitoring reductive elimination of (L4)Ni(CH_2TMS)$_2$ at 50 °C and 60 °C in C_6D_6 8
  Monitoring reductive elimination of (L4)Ni(CH_2TMS)$_2$ at 50 °C, 60 °C and 70 °C in THF-*d*$_8$ 10
  Monitoring reductive elimination of (L4)Ni(CH_2TMS)$_2$ under ambient light, 390 nm irradiation, and darkness 12
  Monitoring reductive elimination of (L4)Ni(CH_2TMS)$_2$ at 60 °C in C_6D_6 with 1.0 equiv. DMF 13
  Monitoring reductive elimination of (L4)Ni(CH_2TMS)$_2$ at 60 °C in C_6D_6 with 1.0 equiv. MA 14
  Monitoring reductive elimination of (L4)Ni(CH_2TMS)$_2$ in CD_3CN with 1.0 equiv. 1-fluoro-2,4,6-trimethylpyridinium tetrafluoroborate 16
S4. UV-VIS, and Cyclic Voltammetry 18
  UV-VIS Spectroscopy 18
  Cyclic Voltammetry 19
S5. NMR Spectra of Synthesized Complexes 20
S6. Crystallographic Data 21
S7. Computational details 26
S8. References 27

S1. General Considerations

Solvents. Reactions were carried out under N_2 in a glovebox or on a Schlenk line, in solvents (THF, Et_2O, toluene) that had been dried and degassed using an Innovative Technologies solvent purification system, then stored under N_2 over 4 Å molecular sieves for at least 16 h prior to use. Pentane was degassed by bubbling with N_2 and stored under N_2 over 4 Å molecular sieves for at least 16 h prior to use. C_6D_6, C_7D_8, THF-d_8, (Eurisotop) were freeze/pump/thaw degassed (4x) and likewise stored under N_2 over 4 Å molecular sieves for at least 16 h prior to use.

Reagents. Bipyridine, neocuproine, and bathocuproine were purchased from Fluorochem. Trimethoxylbenzene (TMB) was purchased from TCI Chemicals. Ni(acac)$_2$, and Ni(COD)$_2$ were purchased from Strem Chemicals. Complexes (py)$_2$Ni(CH$_2$TMS)$_2$,[1] (phen)Ni(CH$_2$TMS)$_2$,[1] (Bathocuproine)$_2$Ni,[2] and ligand 4,7-dimethoxy-2,9-dimethyl-1,10-phenanthroline[3] were synthesized according to literature procedures.

Analytical methods. Flash chromatography was performed with Sigma Aldrich technical grade silica gel 60 (230-400 mesh). Thin layer chromatography was carried out using Merck TLC Silica gel 60 F254. NMR spectra were recorded on Bruker Avance Ultrashield 300, 400, or 500 MHz spectrometers, with chemical shifts reported in parts per million (ppm) and coupling constants, *J*, reported in hertz. Quantitative NMR experiments were performed with d1 set to 10s (^{1}H). Gas chromatographic analyses were performed on an Agilent 6890N gas chromatograph with an FID detector.

S2. Synthesis and Characterization of Complexes

Synthesis of (L4)Ni(CH_2TMS)$_2$

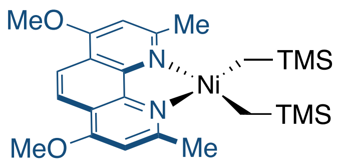

In the glovebox, (py)$_2$Ni(CH_2TMS)$_2$ (150 mg, 0.38 mmol) was added to a 12 mL vial. A stir bar was added, and the vial was charged with 3 mL of toluene and cooled to -36 °C. To this cooled solution, 4,7-dimethoxy-2,9-dimethyl-1,10-phenanthroline (103 mg, 0.38 mmol, 1 equiv) was added as a solid with 1 mL of cooled toluene (-36 °C). Upon addition of L4, a rapid colour change from red to purple was observed and the solvent was removed after 10 minutes. The solid was then redissolved in Et_2O and filtered through a celite plug with a black solid being filtered off and a purple solution collected. The solvent was then removed to afford a purple solid and washed with cold pentane (1 mL x 3, -36 °C) to give (L4)Ni(CH_2TMS)$_2$ (116 mg, 68 % yield) as a purple powder.

Stability: Unstable in air
^{1}H NMR (400 MHz, C_6D_6) δ 7.97 (s, 2H), 6.05 (s, 2H), 3.06 (s, 6H), 2.79 (s, 6H), 0.55 (s, 18H), -0.15 (s, 4H)**.** **^{13}C NMR** (101 MHz, Tol-*d$_8$*) δ 162.6, 160.8, 146.9, 119.6, 118.4, 105.9, 55.2, 28.0, 4.0, -0.0.
UV-VIS (nm) = 339 nm, 515 nm
Cyclic Voltammetry = E_p value for Ni(II)/Ni(III) = -0.40 V vs SCE (THF, 100 mM ^{n}Bu$_4$PF$_6$). For comparison to unsubstituted (phen)Ni(CH_2TMS)$_2$, the E_p value for Ni(II)/Ni(III) = 0.22 V vs SCE (THF, 100 mM ^{n}Bu$_4$PF$_6$).
Single Crystal XRD: Slow evaporation in Et_2O at -36 °C.

S3. Stochiometric Reactions.

Instability of (Bc)Ni(CH_2TMS)$_2$ and formation of (Bc)$_2$Ni

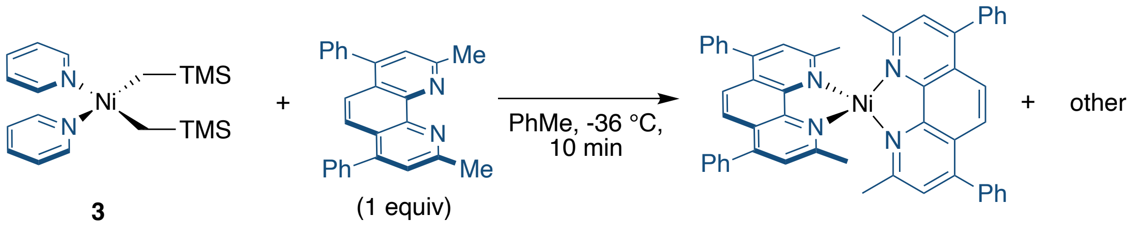

Monitored by ^{1}H NMR. In the glovebox, (py)$_2$Ni(CH_2TMS)$_2$ (32 mg, 0.08 mmol) was added to a 12 mL vial. A stir bar was added, and the vial was charged with 2 mL of toluene and cooled to -36 °C. To this cooled solution, bathocuproine (30 mg, 0.08 mmol, 1 equiv) was added as a solid with 1 mL of cooled toluene (-36 °C). Upon addition of bathocuproine, a rapid colour change from red to purple was observed and the solvent was removed after 10 minutes. A portion of this solid (ca. 2 mg) was then redissolved in C_6D_6 and analyzed by ^{1}H NMR which identified (Bc)2Ni as the major bathocuproine containing product (Figure S1, bottom spectrum – t_1). The remaining solid was dissolved in Et_2O and filtered through a celite plug with a black solid being filtered off and a purple solution collected. The solvent was then removed to afford a purple solid which was redissolved in C_6D_6 and analyzed by ^{1}H NMR which again identified (Bc_2Ni) as the major bathocuproine containing product.

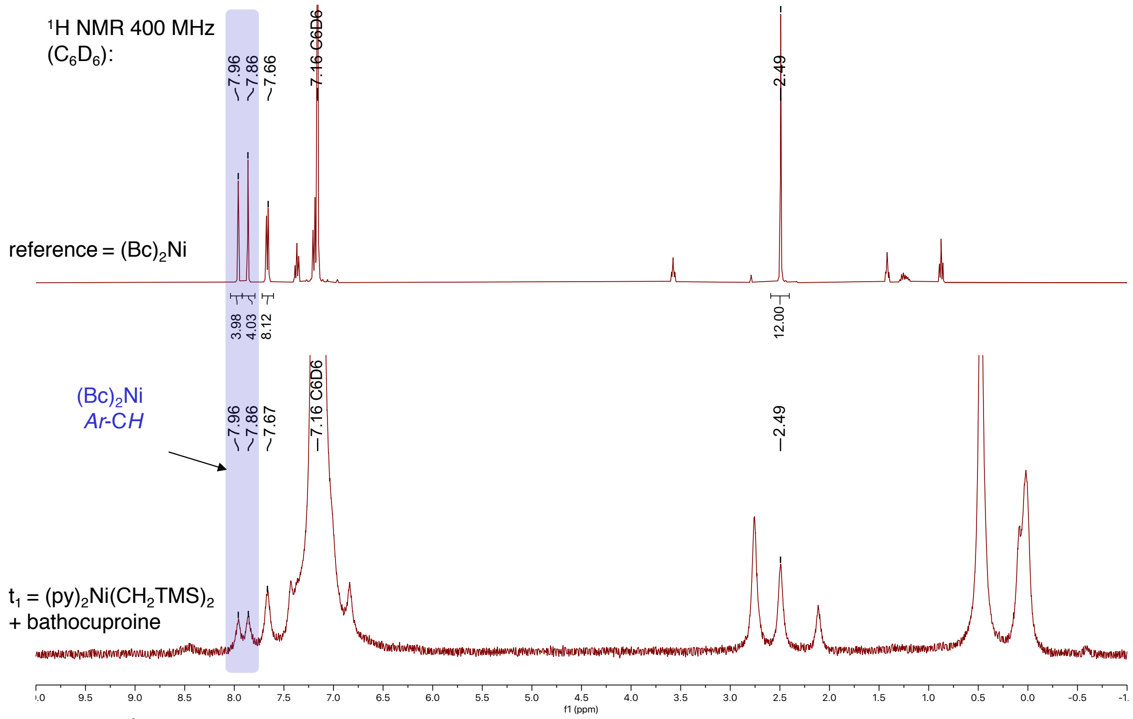

Figure S1. ^{1}H NMR spectra of the crude reaction between (py)$_2$Ni(CH_2TMS)$_2$ and bathocuproine to form (Bc)$_2$Ni. (Bc)$_2$Ni (blue), Bc = bathocuproine.

Attempting analogous synthesis to (bipy)$NiEt_2$ with neocuproine

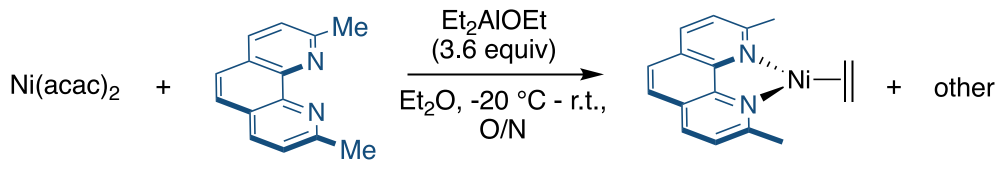

On the Schlenk line, $Ni(acac)_2$ (208 mg, 0.66 mmol) and neocuproine (267 mg, 1.28 mmol, 2 equiv) was added to an oven dried 25 mL RBF under N_2. A stir bar was added, and the flask was charged with 8 mL of Et_2O and cooled to -20 °C. To this cooled solution, a 1.3 M solution of $Et_2Al(OEt)$ in hexane (1.8 ml, 2.34 mmol, 3.6 equiv) was added dropwise and let stir overnight. The RBF was then brought into the glovebox and filtered, with the filtrate left to crystallize in the fridge at -36 °C. After 24 h, the crystalline material was filtered and washed with pentane (1 ml x 3). Single crystal XRD of crystals unambiguously identified the structure (neo)$Ni(C_2H_4)$ had formed in 122 mg (60 % yield) of solid material. Unexpectedly, ^{1}H NMR of the crystalline material (neo)$Ni(C_2H_4)$ was NMR silent, suggesting (neo)$Ni(C_2H_4)$ is either insoluble in a variety of solvents tested or paramagnetic due to significant back donation into ethylene and may be better described as a Ni(I) species. In no solvent did we observe paramagnetic NMR signals (expanded sweep width of 200 to -200 ppm).

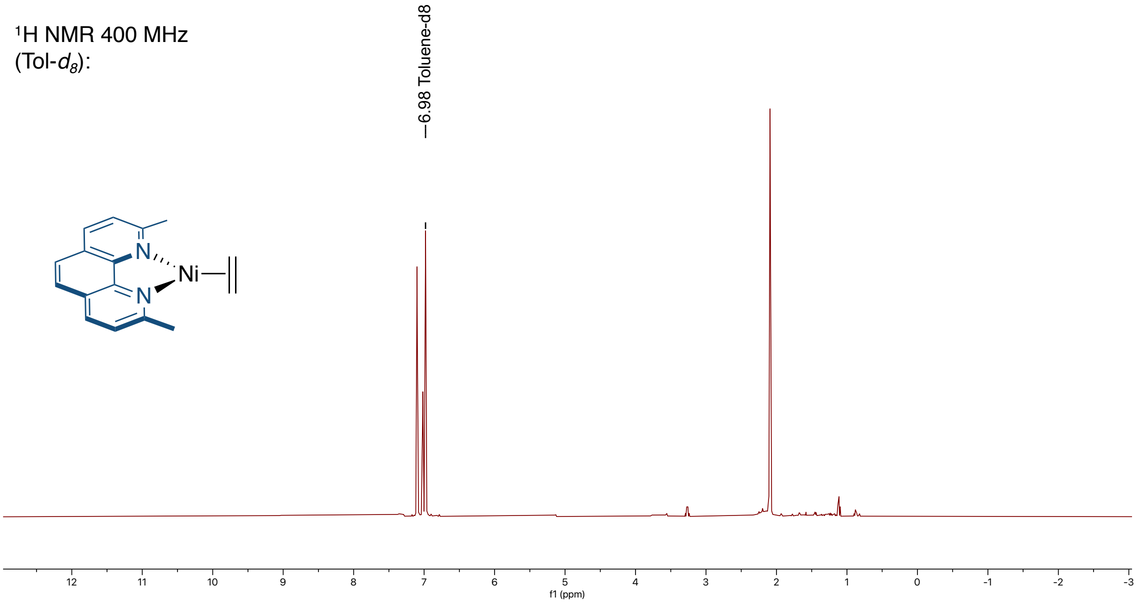

Figure S2. ^{1}H NMR (Toluene-d_8) spectra of crystalline material (neocuproine)$Ni(C_2H_4)$. Neo = neocuproine.

Reductive elimination of (Phen)Ni(CH2TMS)2

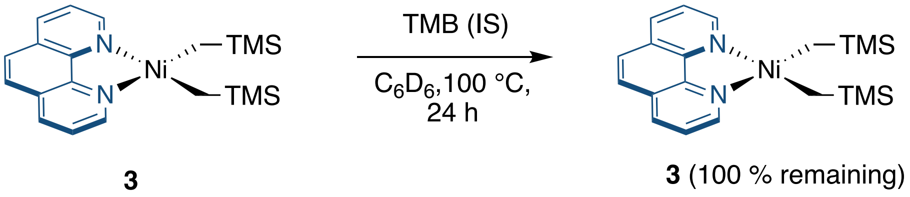

Monitored by ^{1}H NMR. In the glovebox, (phen)Ni(CH_2TMS)$_2$ (7.8 mg, 0.02 mmol) was added to a 4 mL vial with TMB (2.1 mg, internal standard). The vial was charged with 1 mL of C_6D_6 and filtered through a celite plug and transferred to a J-Young NMR tube where the integral ratio of TMB and (phen)Ni(CH_2TMS)$_2$ was measured (Figure S3 – bottom – t_0). The J-young tube was then transferred to a preheated oil bath and heated at 100 °C before the integral ratio of TMB and (phen)Ni(CH_2TMS)$_2$ was measured again (Figure S3 – top – t_1) in which no conversion of (phen)Ni(CH_2TMS)$_2$ was observed.

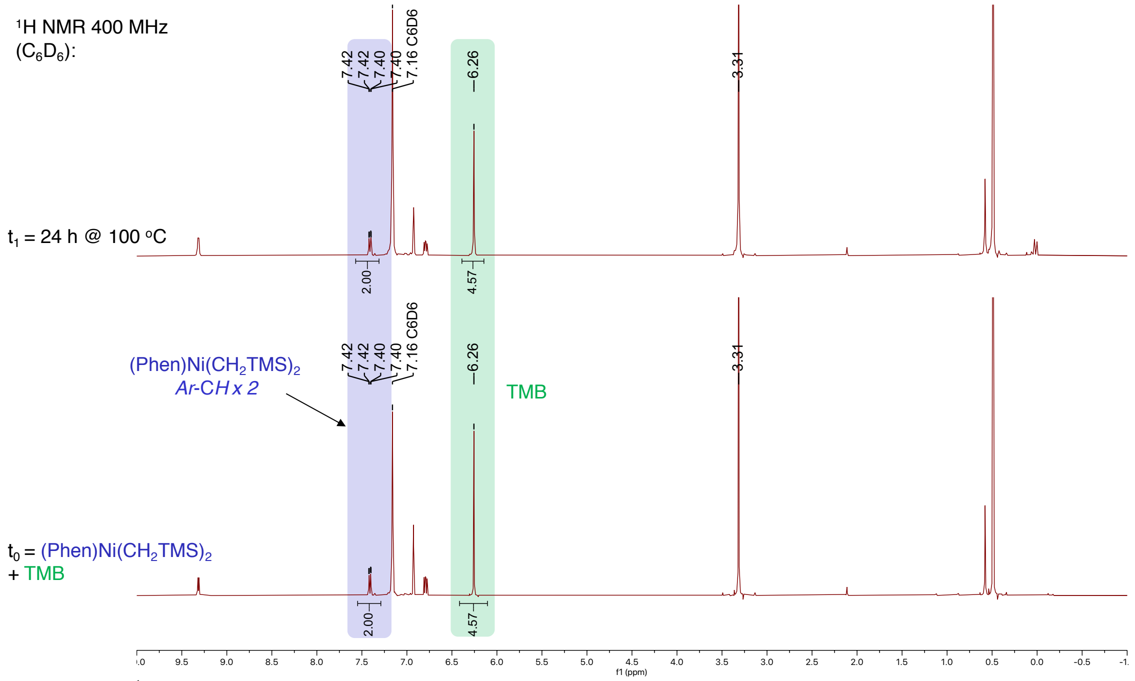

Figure S3. ^{1}H NMR (C_6D_6) spectra of monitoring the thermolysis of (Phen)Ni(CH_2TMS)$_2$ with TMB as an internal standard. (Phen)Ni(CH_2TMS)$_2$, blue. TMB, green.

Independent synthesis of $(L4)_2Ni$

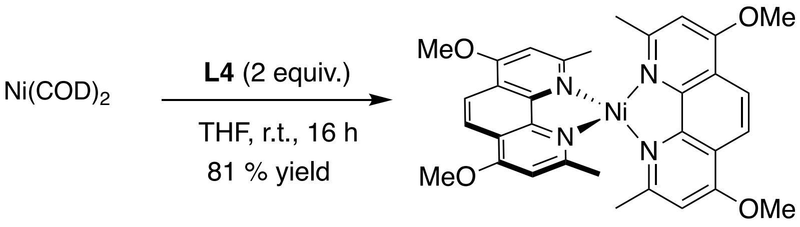

In the glovebox, $Ni(COD)_2$ (6.9 mg, 0.03 mmol) and L4 (13.5 mg, 0.05 mmol) were added to a 12 mL vial with a stirbar. The vial was charged with 5 mL of THF and let stir for 16 h, in which a black suspension was formed. The solvent was decanted and the remaining solid washed with Et_2O (2 mL x 2) and toluene (2 mL x 2) to afford $(L4)_2Ni$ as a black solid (12.0 mg, 81 % yield). Due to the very poor solubility of $(L4)_2Ni$ in all solvents tested, it was difficult to obtain good spectroscopic data, even with saturated solutions. No carbon signals were visible with high scan numbers of 10000. This insolubility of $(L4)_2Ni$ also made crystallographic characterization challenging and after extensive attempts, we were unsuccessful in confirming the structure. While we were unable to obtain single crystals of $(L4)_2Ni$, single crystal XRD of a synthesis of $(L4)_2Ni$ using an impure batch of L4 (contained 7-methoxy-2,9-dimethyl-1,10-phenanthrolin-4-ol) afforded poor quality crystals of the analogous complex (L4)(7-methoxy-2,9-dimethyl-1,10-phenanthrolin-4-ol)Ni^0.

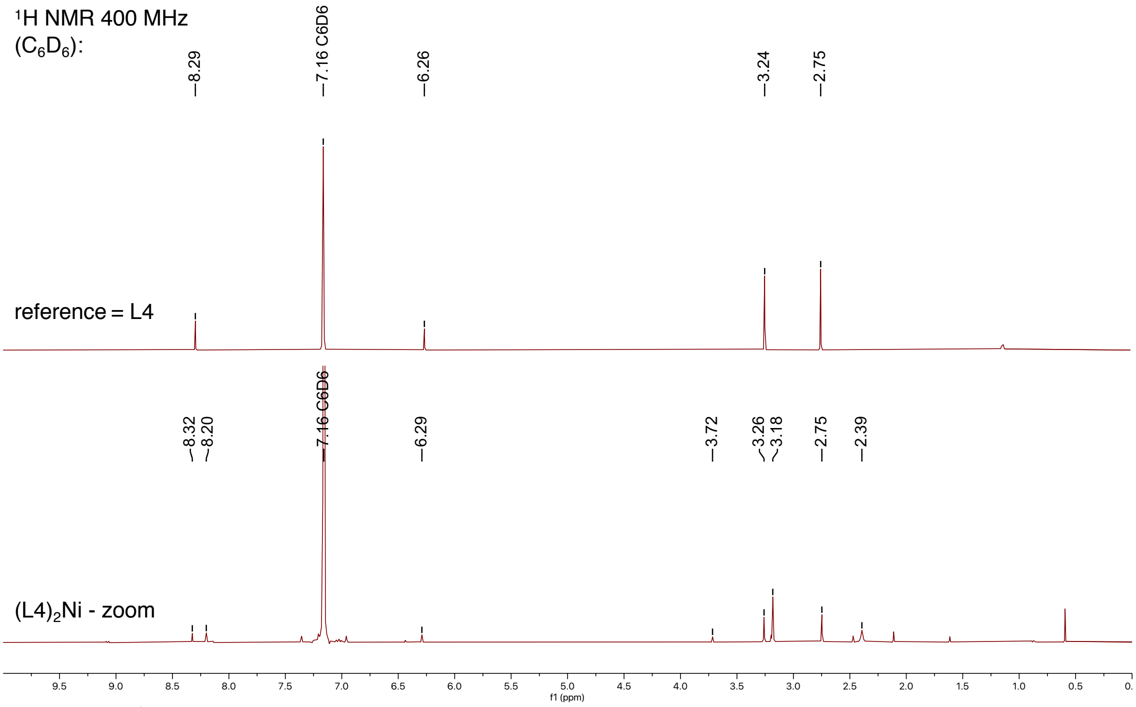

Figure S4. ^1H NMR (C_6D_6) spectra of $(L4)_2Ni$.

Monitoring reductive elimination of (L4)Ni(CH_2TMS)$_2$ at 50 °C and 60 °C in C_6D_6

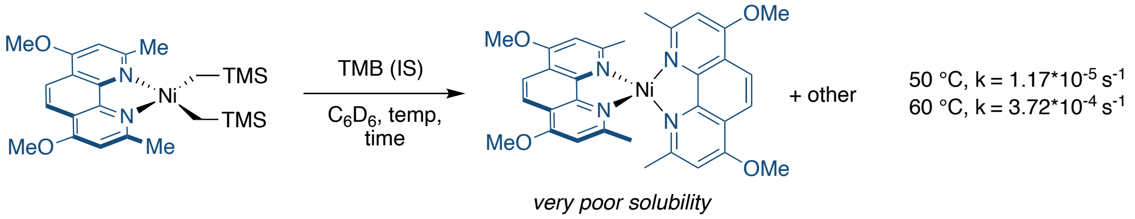

Monitored by quantitative ^{1}H NMR. In the glovebox, (L4)Ni(CH_2TMS)$_2$ (16.4 mg, 0.035 mmol) and TMB (7 mg, internal standard) were added to a 4-dram vial with 3.2 mL of C_6D_6. The dark mixture was stirred vigorously for 10 minutes, and was filtered through an HPLC filter into a new 4-dram vial. Two aliquots (0.6 mL each) were then taken and each were added to separate J-Young NMR tubes and the initial integration of (L4)Ni(CH_2TMS)$_2$ and TMB was recorded. The J-Young NMR tubes were then analyzed by ^{1}H NMR for the disappearance of (L4)Ni(CH_2TMS)$_2$ over time at the specified temperatures of 50 °C and 60 °C respectively. **Note:** The solution of (L4)Ni(CH_2TMS)$_2$ and TMB was filtered as an extra precaution to remove any trace undissolved TMB or (L4)Ni(CH_2TMS)$_2$.

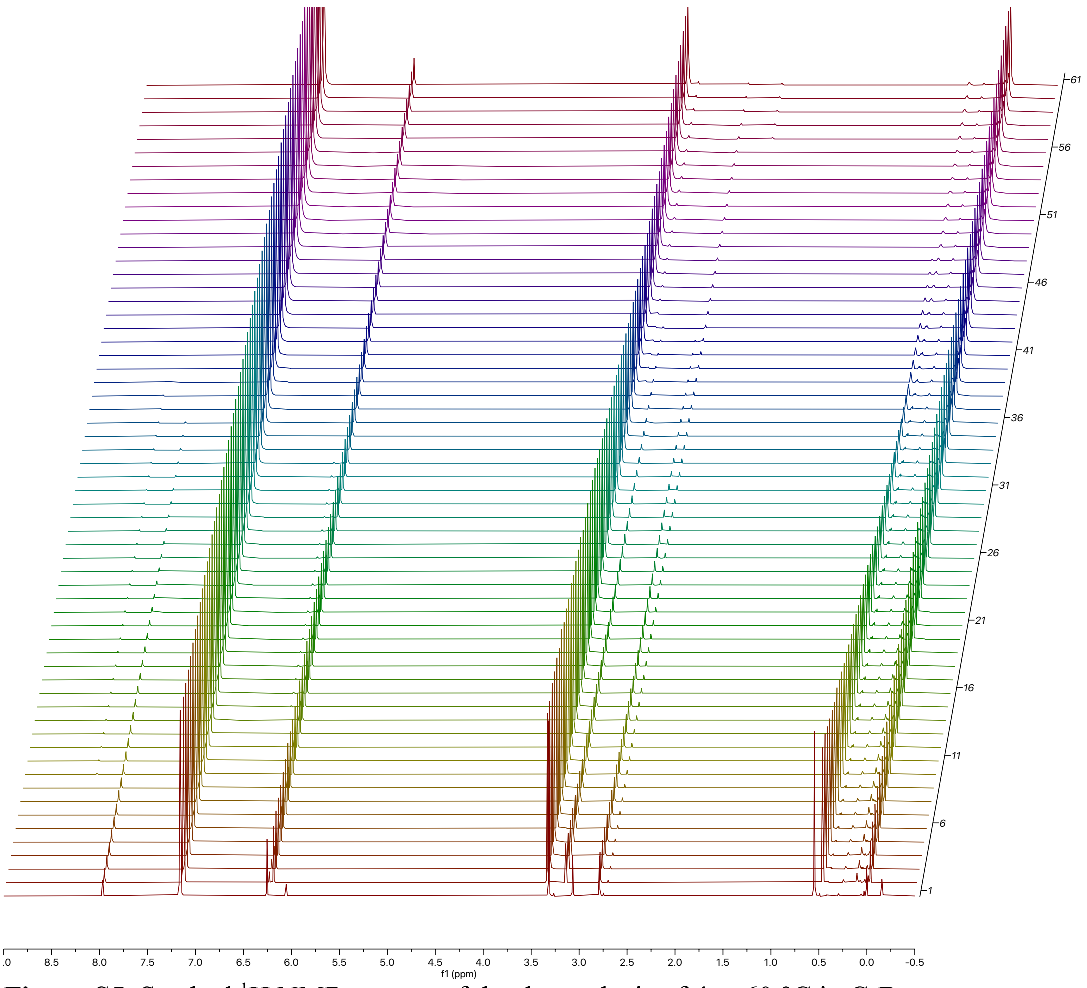

Figure S5. Stacked ^{1}H NMR spectra of the thermolysis of **4** at 60 °C in C_6D_6.

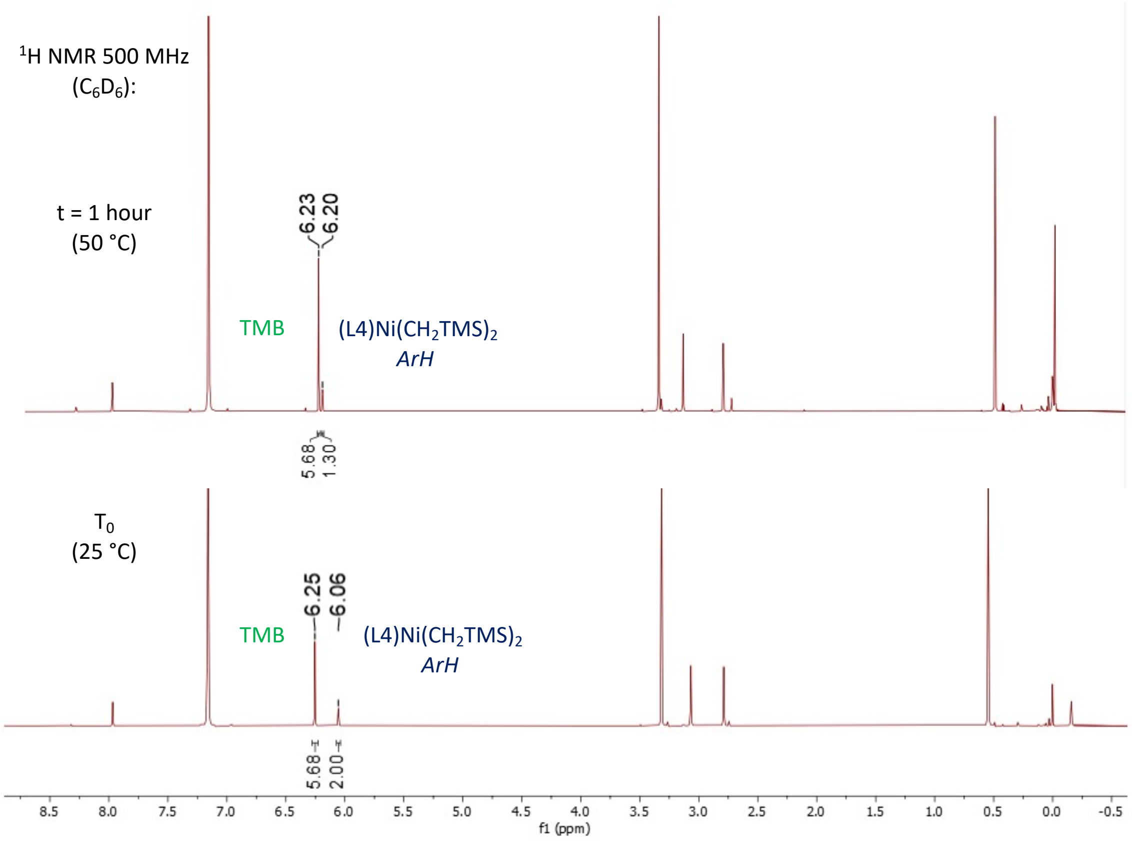

Figure S6. Example quantitative ^{1}H NMR spectra of TMB and (L4)Ni(CH_2TMS)$_2$ monitoring consumption of (L4)Ni(CH_2TMS)$_2$ over time. Internal standard trimethoxybenzene, TMB (green) and (L4)Ni(CH_2TMS)$_2$ (blue).

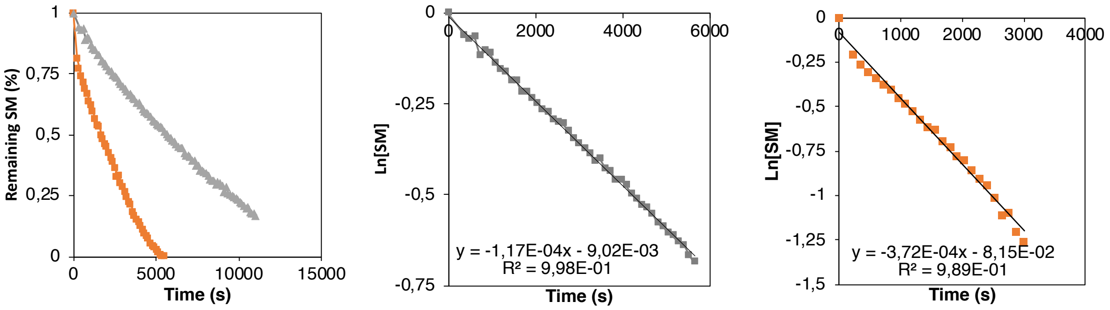

Figure S7. Plotted graphs of the thermolysis of **4** at 50 °C (grey), and 60 °C (orange) in C_6D_6.

Monitoring reductive elimination of (L4)Ni(CH_2TMS)$_2$ at 50 °C, 60 °C and 70 °C in THF-d_8

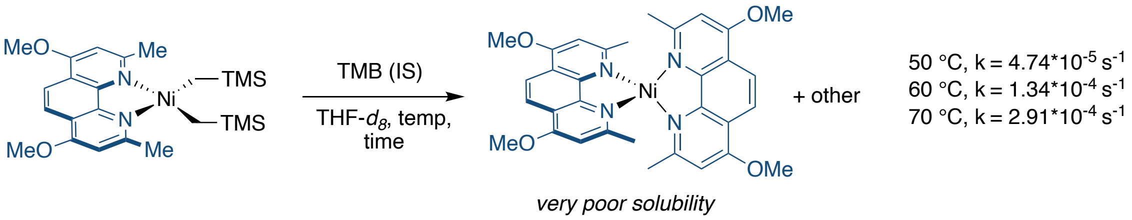

Monitored by quantitative ^{1}H NMR. In the glovebox, (L4)Ni(CH_2TMS)$_2$ (13.5 mg, 0.029 mmol) and TMB (5 mg, internal standard) were added to a 4-dram vial with 3.2 mL of THF-d_8. The dark mixture was stirred vigorously for 10 minutes, and was filtered through an HPLC filter into a new 4-dram vial. Three aliquots (0.6 mL each) were then taken and each were added to separate J-Young NMR tubes and the initial integration of (L4)Ni(CH_2TMS)$_2$ and TMB was recorded. The J-Young NMR tubes were then analyzed by ^{1}H NMR for the disappearance of (L4)Ni(CH_2TMS)$_2$ over time at the specified temperatures of 50 °C, 60 °C and 70 °C respectively. **Note:** Due to the delay upon inserting the NMR tube, locking, and shimming before measurement there is some error in the first data point which results in a larger than anticipated initial slope.

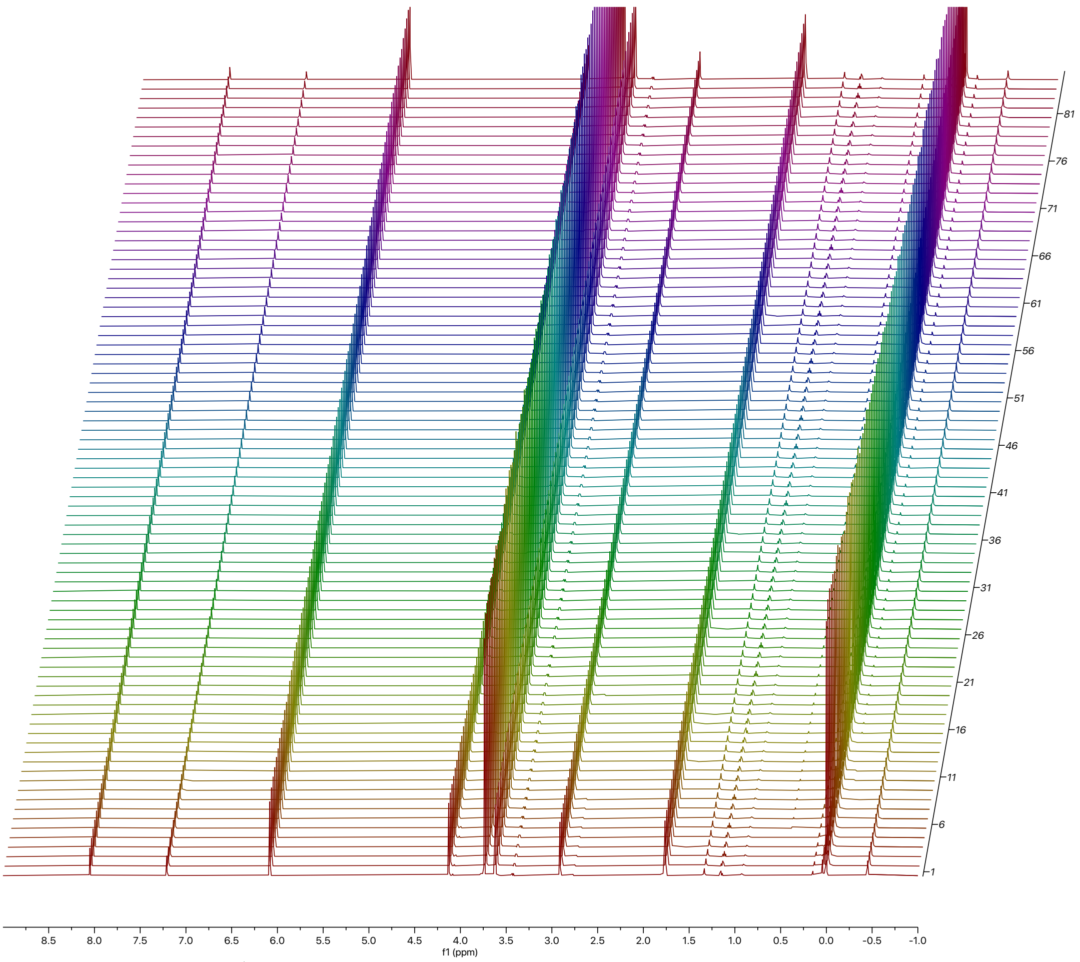

Figure S8. Stacked ^{1}H NMR spectra of the thermolysis of **4** at 50 °C in THF-d_8.

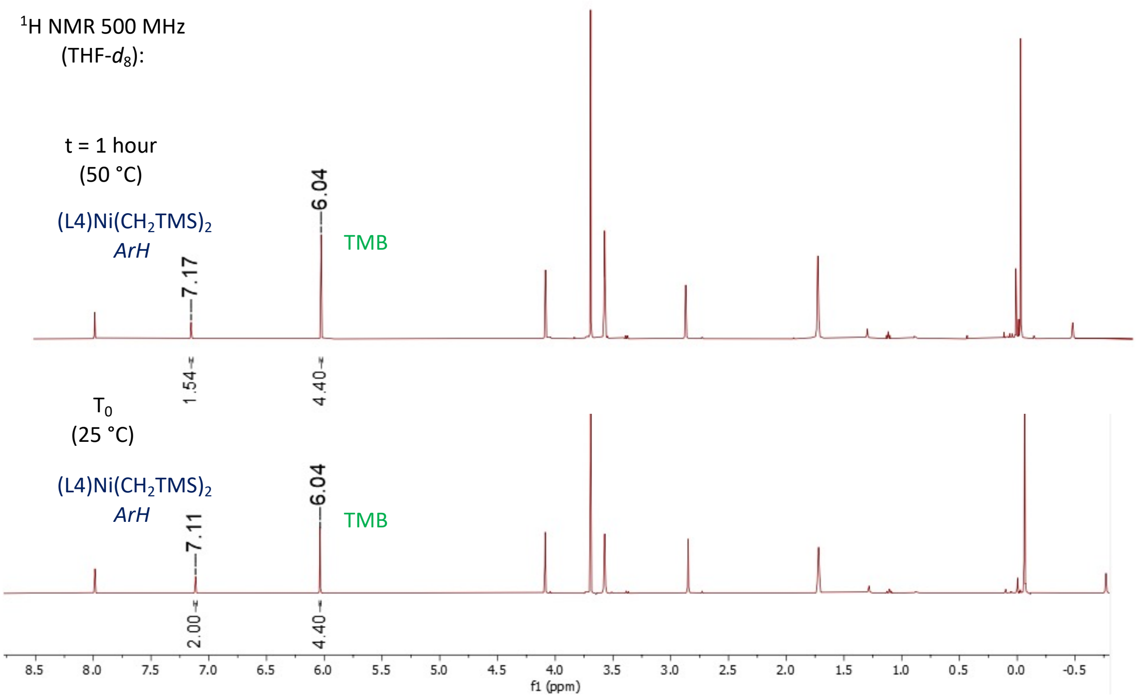

Figure S9. Example quantitative ^{1}H NMR spectra of TMB and (L4)Ni($CH_2TMS)_2$ monitoring consumption of (L4)Ni($CH_2TMS)_2$ over time. Internal standard trimethoxybenzene, TMB (green) and (L4)Ni($CH_2TMS)_2$ (blue).

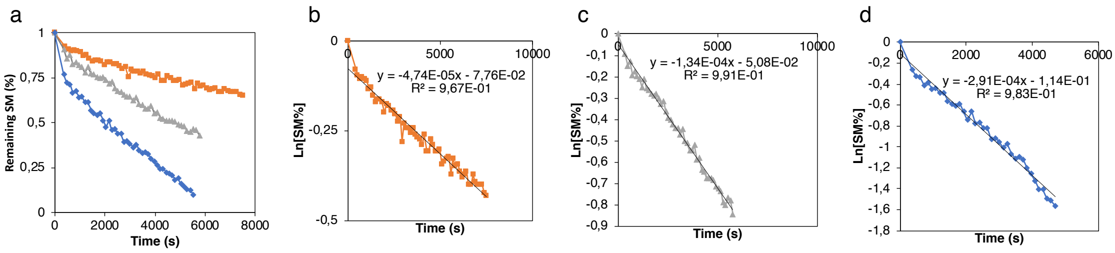

Figure S10. Plotted graphs of the thermolysis of **4** at 50 °C (orange), 60 °C (grey), and 70°C (blue) in THF-*d*8.

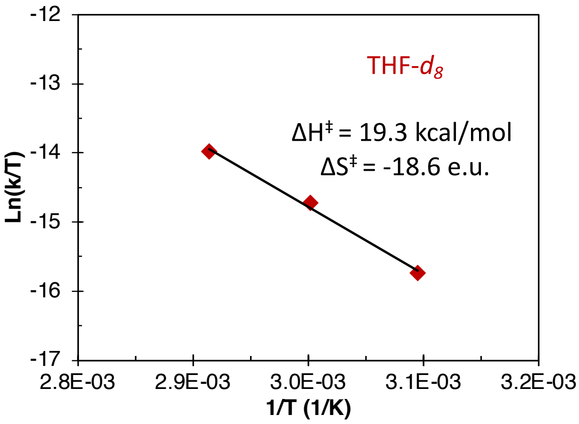

Figure S11. Eyring plot analysis in THF-*d*8 between 50 and 70°C.

Monitoring reductive elimination of (L4)Ni(CH_2TMS)$_2$ under ambient light, 390 nm irradiation, and darkness

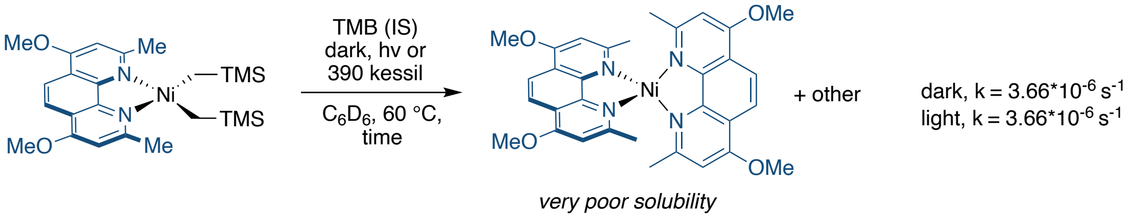

Monitored by quantitative ^{1}H NMR. In the glovebox, (L4)Ni(CH_2TMS)$_2$ (14.2 mg, 0.030 mmol) and TMB (5 mg, internal standard) were added to a 4-dram vial with 3.0 mL of toluene-d_8. The dark mixture was stirred vigorously for 10 minutes, and was filtered through an HPLC filter into a new 4-dram vial. Three aliquots (0.6 mL each) were then taken and each were added to separate J-Young NMR tubes and the initial integration of (L4)Ni(CH_2TMS)$_2$ and TMB was recorded. The J-Young NMR tubes were then analyzed by ^{1}H NMR for the disappearance of (L4)Ni(CH_2TMS)$_2$ over time under each of the specified conditions (ambient light, 390 nm Kessil LED irradiation, darkness). **Note:** The temperature under 390 nm Kessil LED irradiation could not accurately be controlled and we suspect the heating from the Kessil lamp is the reason for the increase in reaction rate.

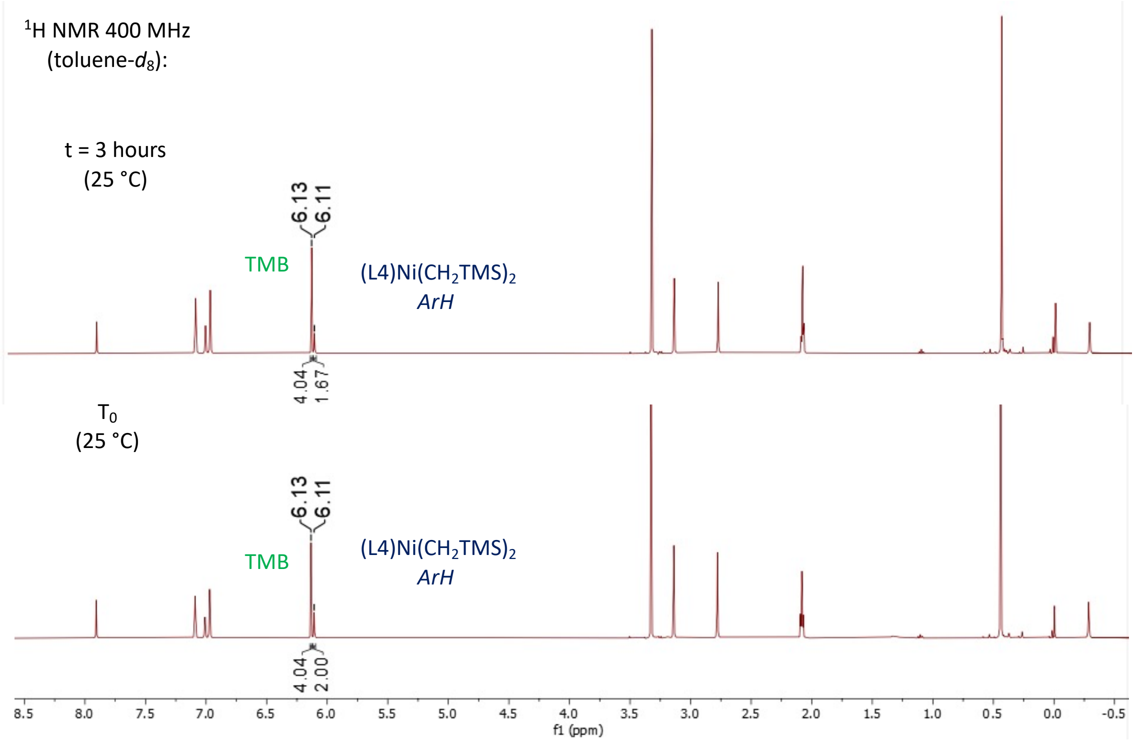

Figure S12. Example quantitative ^{1}H NMR spectra of TMB and (L4)Ni(CH_2TMS)$_2$ monitoring consumption of (L4)Ni(CH_2TMS)$_2$ over time. Internal standard trimethoxybenzene, TMB (green) and (L4)Ni(CH_2TMS)$_2$ (blue).

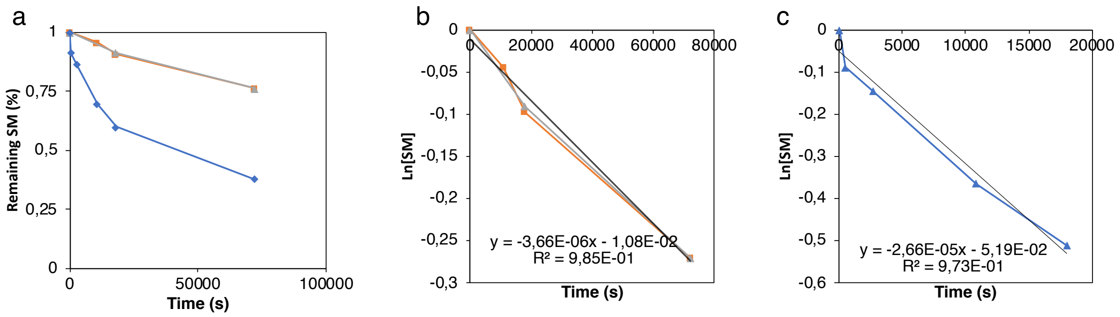

Figure S13. Plotted graphs of the thermolysis of **4** under ambient light (orange), in the dark (grey), at room temperature and under 390 nm kessil irradiation (blue) at elevated temperature in toluene-d_8.

Monitoring reductive elimination of (L4)Ni(CH_2TMS)$_2$ at 60 °C in C_6D_6 with 1.0 equiv. DMF

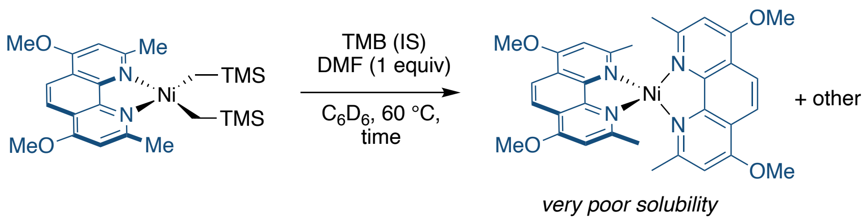

k = 1.16*10^{-4} s^{-1}

Monitored by quantitative ^{1}H NMR. In the glovebox, (L4)Ni(CH_2TMS)$_2$ (14.2 mg, 0.030 mmol) and TMB (6 mg, internal standard) were added to a 4-dram vial with 3.0 mL of C_6D_6. The dark mixture was stirred vigorously for 10 minutes, and was filtered through an HPLC filter into a new 4-dram vial. Two aliquots (0.6 mL each) were then taken and each were added to separate J-Young NMR tubes and the initial integration of (L4)Ni(CH_2TMS)$_2$ and TMB was recorded. To the remaining J-Young NMR tube was added 1.0 equiv of DMF. This J-Young NMR tube was then analyzed by ^{1}H NMR for the disappearance of (L4)Ni(CH_2TMS)$_2$ over time at the specified temperature of 60 °C.

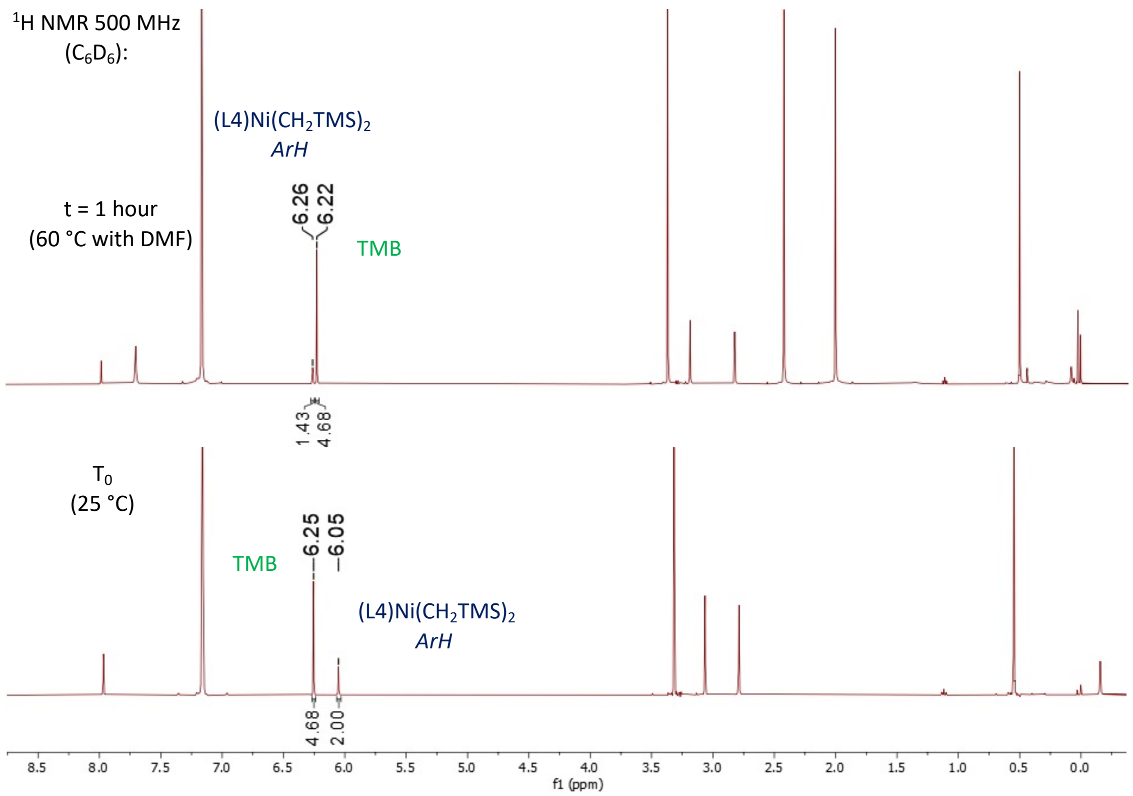

Figure S14. Example quantitative ^{1}H NMR spectra of TMB and (L4)Ni(CH_2TMS)$_2$ monitoring consumption of (L4)Ni(CH_2TMS)$_2$ over time with DMF additive. Internal standard trimethoxybenzene, TMB (green) and (L4)Ni(CH_2TMS)$_2$ (blue).

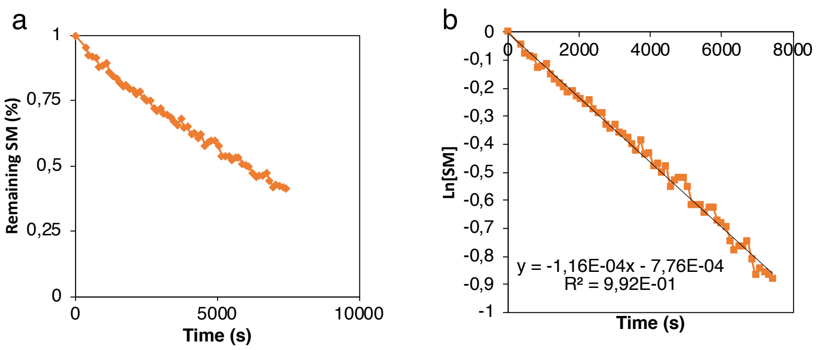

Figure S15. Plotted graphs of the thermolysis of **4** with 1 equiv of DMF added at 60 °C in C_6D_6.

Monitoring reductive elimination of (L4)Ni(CH_2TMS)$_2$ at 60 °C in C_6D_6 with 1.0 equiv. methyl acrylate (MA)

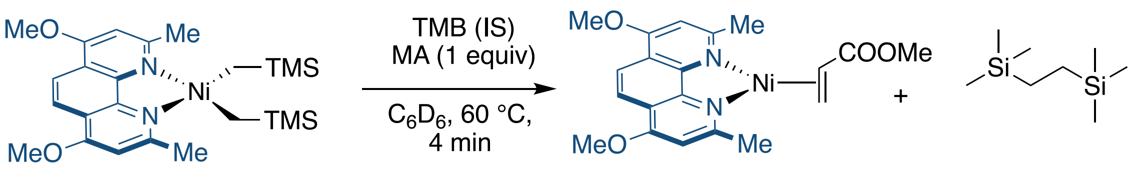

5 *proposed* quantitative

Monitored by quantitative ^{1}H NMR. In the glovebox, (L4)Ni(CH_2TMS)$_2$ (14.2 mg, 0.030 mmol) and TMB (6 mg, internal standard) were added to a 4-dram vial with 3.0 mL of C_6D_6. The dark mixture was stirred vigorously for 10 minutes, and was filtered through an HPLC filter into

a new 4-dram vial. Two aliquots (0.6 mL each) were then taken and each were added to separate J-Young NMR tubes and the initial integration of (L4)Ni(CH_2TMS)$_2$ and TMB was recorded. To the remaining J-Young NMR tube was added 1.0 equiv of MA. This J-Young NMR tube was then analyzed by ^{1}H NMR for the disappearance of (L4)Ni(CH_2TMS)$_2$ over time at the specified temperature of 60 °C. The first time point measured at 4 minutes revealed all of (L4)Ni(CH_2TMS)$_2$ was consumed in the reaction (Figure S16 – middle spectrum – t_1). Monitoring the reaction for 30 minutes longer revealed, no change in the ^{1}H NMR spectra. Comparison of the spectrum at 4 minutes to a reference spectrum of (L4)Ni(CH_2TMS)$_2$ heated at 60 °C (Figure S16 – top spectrum) reveals all the peaks have shifted, with the most notable being the ^{1}H NMR peak at 2.82 ppm. Quantification of the organic products by GC-FID also confirmed quantitative formation of 1,2-bis(trimethylsilyl)ethane. Broad signals at 5.81 ppm and 5.21 ppm for free methacrylate were also observed supporting equilibrium binding of the olefin. Together, this analysis supports the reductive elimination of (L4)Ni(CH_2TMS)$_2$ to form (L4)Ni(MA) and 1,2-bis(trimethylsilyl)ethane.

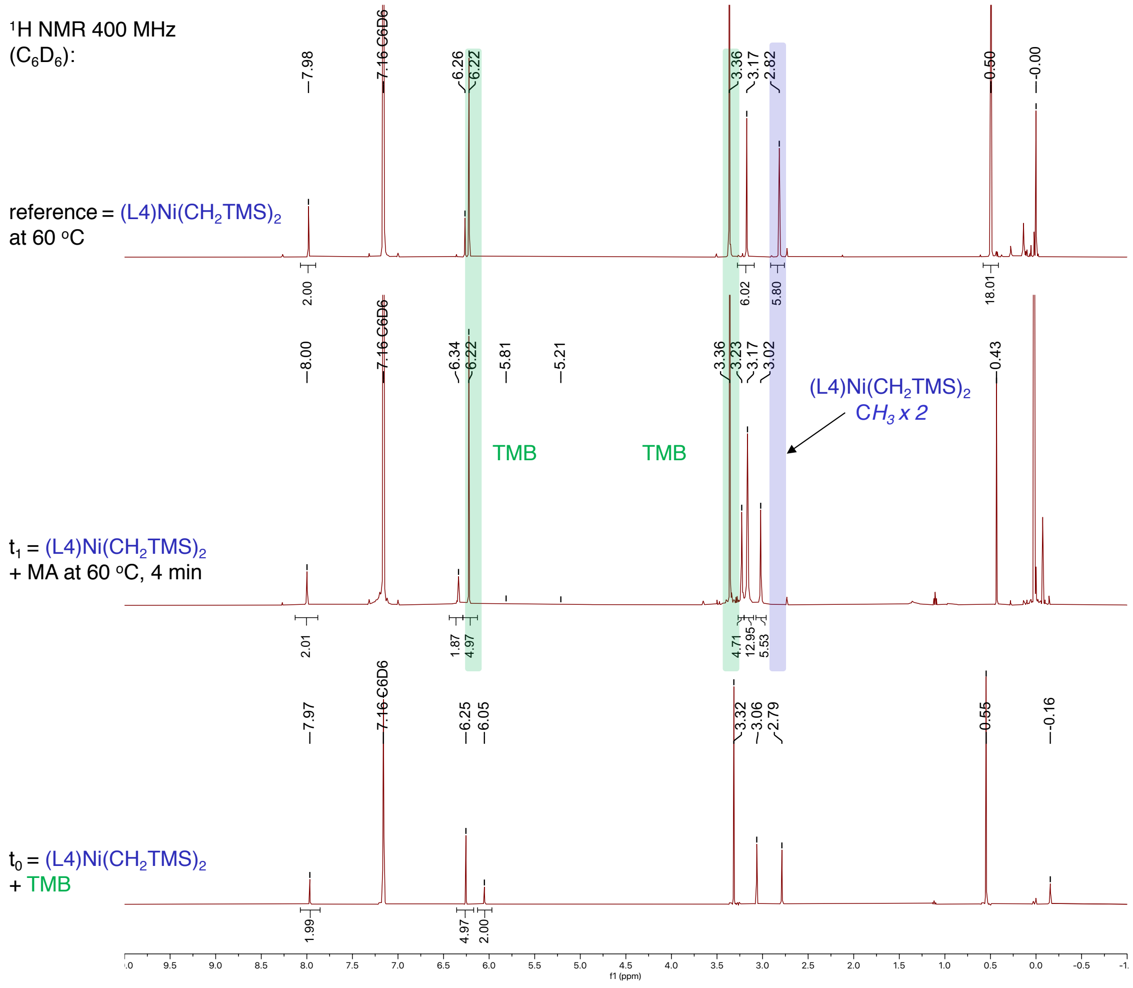

Figure S16. Example quantitative ^{1}H NMR spectra of TMB and (L4)Ni(CH_2TMS)$_2$ monitoring consumption of (L4)Ni(CH_2TMS)$_2$ over time with DMF additive. Internal standard trimethoxybenzene, TMB (green) and (L4)Ni(CH_2TMS)$_2$ (blue).

Monitoring reductive elimination of (L4)Ni(CH_2TMS)$_2$ in CD_3CN with 1.0 equiv. 1-fluoro-2,4,6-trimethylpyridinium tetrafluoroborate

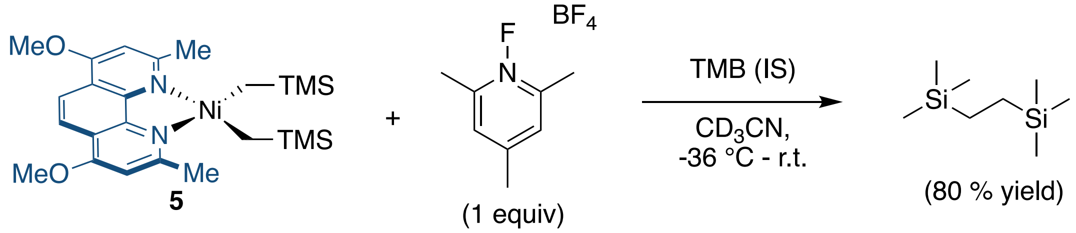

Monitored by ^{1}H NMR. In the glovebox, (L4)Ni(CH_2TMS)$_2$ (4.7 mg, 0.010 mmol) and TMB (3 mg, internal standard) were added to a 4-dram vial with 1.0 mL of CD_3CN. The dark mixture was stirred vigorously for 10 minutes, after which a 0.6 mL aliquot was taken and added to a J-Young NMR tube which was then submitted for ^{1}H NMR analysis. The J-Young NMR tube was then cycled back into the glovebox and was recombined in the vial which contained the initial mixture and was then placed in the freezer to chill at – 36 °C. Following 10 minutes, 0.7 mL of the chilled mixture was then added to a vial containing pre-weighed 1-fluoro-2,4,6-trimethylpyridinium tetrafluoroborate (1.6 mg, 1.0 equiv.) and this mixture was subsequently added to a J-Young NMR tube which was then submitted for ^{1}H NMR analysis immediately in which no (L4)Ni(CH_2TMS)$_2$ remained (figure S17 – top spectrum – t_1) and analysis by paramagnetic ^{1}H NMR identified new signals (figure S18). The mixture was then analyzed by GC-FID to quantify the formation of 1,2-bis(trimethylsilyl)ethane in 80 % yield.

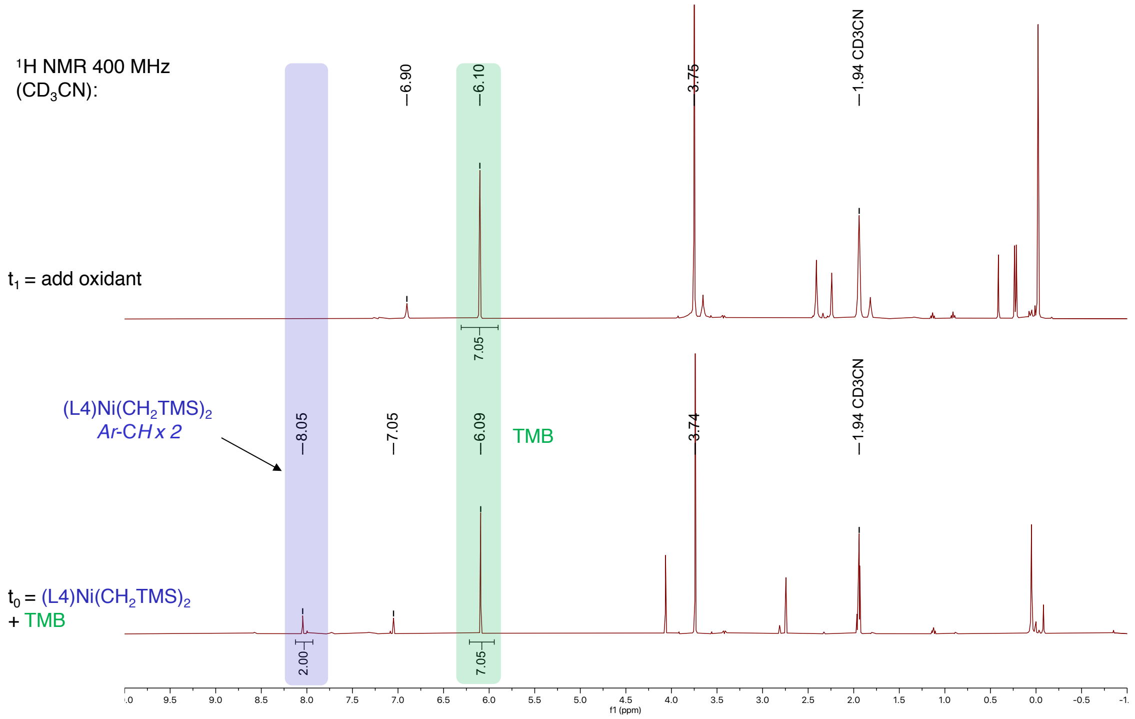

Figure S17. ^{1}H NMR spectra of TMB and (L4)Ni(CH_2TMS)$_2$ monitoring consumption of (L4)Ni(CH_2TMS)$_2$ after the addition of 1-Fluoro-2,4,6-trimethylpyridinium tetrafluoroborate. Internal standard trimethoxybenzene, TMB (green) and (L4)Ni(CH_2TMS)$_2$ (blue).

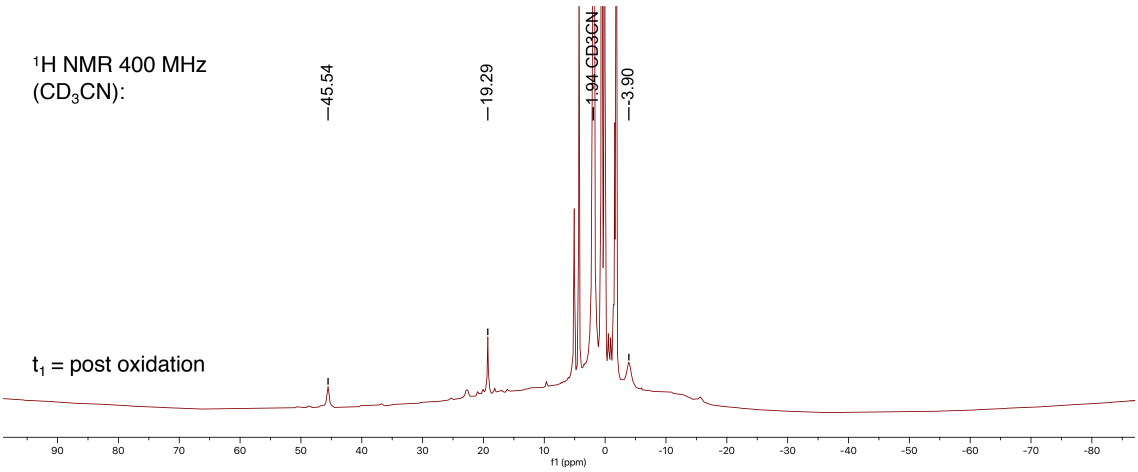

Figure S18. Paramagnetic ^{1}H NMR of the crude reaction mixture post oxidation.

S4. UV-VIS, and Cyclic Voltammetry

UV-VIS Spectroscopy

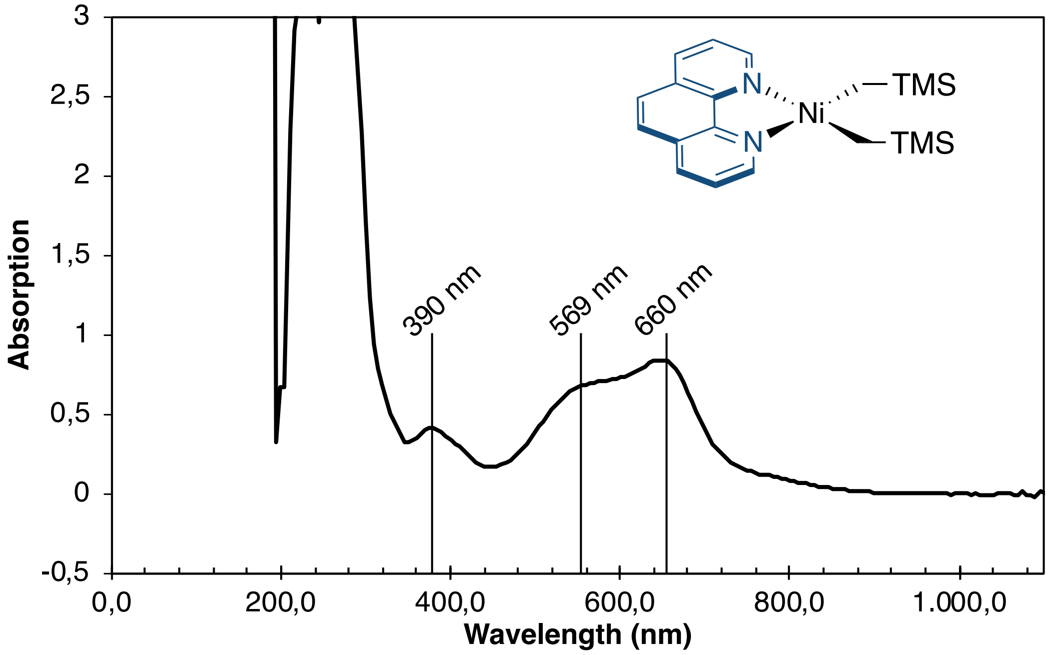

Figure S19. UV-VIS spectra of (phen)Ni(CH_2TMS)$_2$ in THF (0.3 mM).

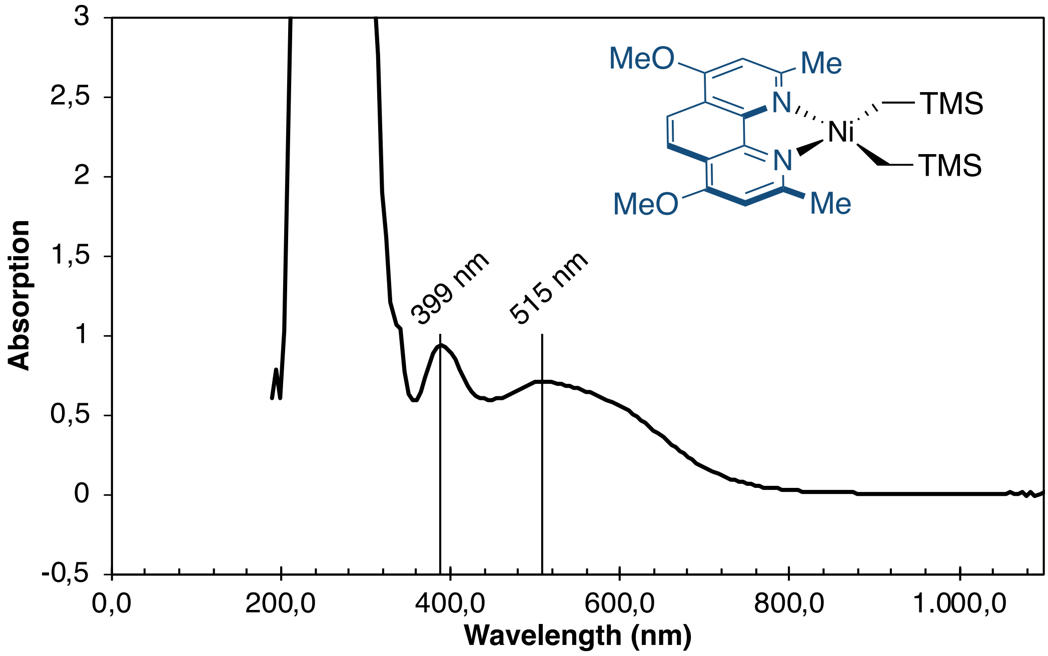

Figure S20. UV-VIS spectra of (4,7-dimethoxy-2,9-dimethyl-1,10-phenanthroline) Ni(CH_2TMS)$_2$ in THF (0.3 mM)

Cyclic Voltammetry

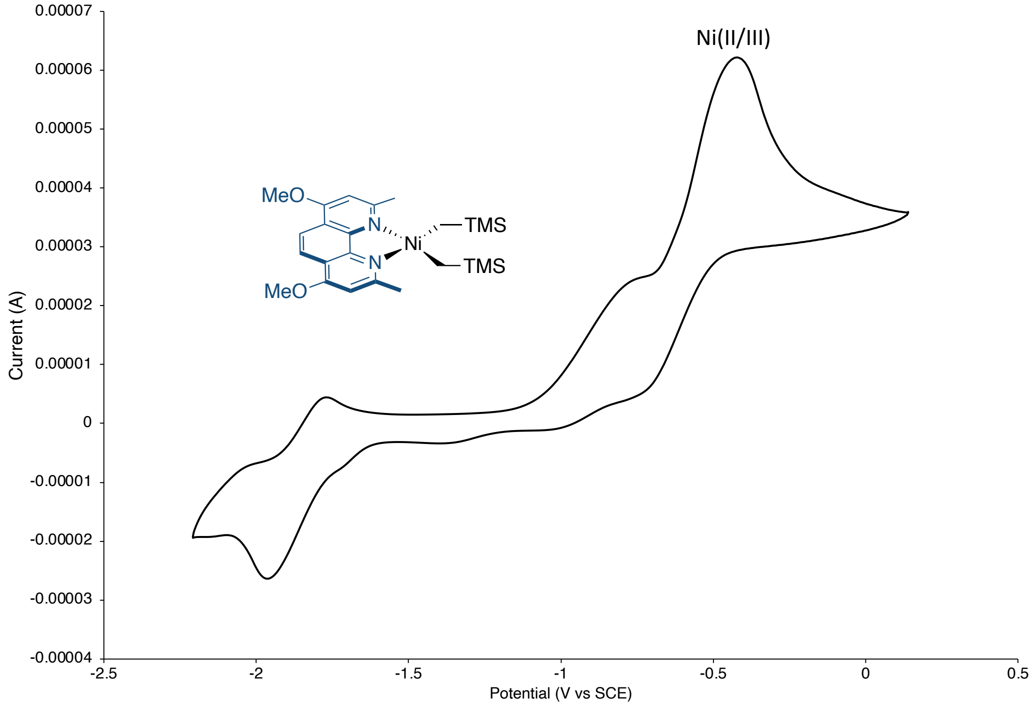

Figure S21. Cyclic voltammogram of (L4)Ni(CH_2TMS)$_2$. Voltammograms were taken using a glassy carbon working electrode in a 0.1 M [$^nBu_4N$][PF_6] supporting electrolyte THF solution with a 100 mV/s scan rate and 0.01 M of sample referenced to Fc (+0.38 V vs SCE). Scans were started at the open-circuit potential and scanned in the anode direction first. E_p value for (L4)Ni(CH_2TMS)$_2$ Ni(II)/Ni(III) = -0.40 V vs SCE.

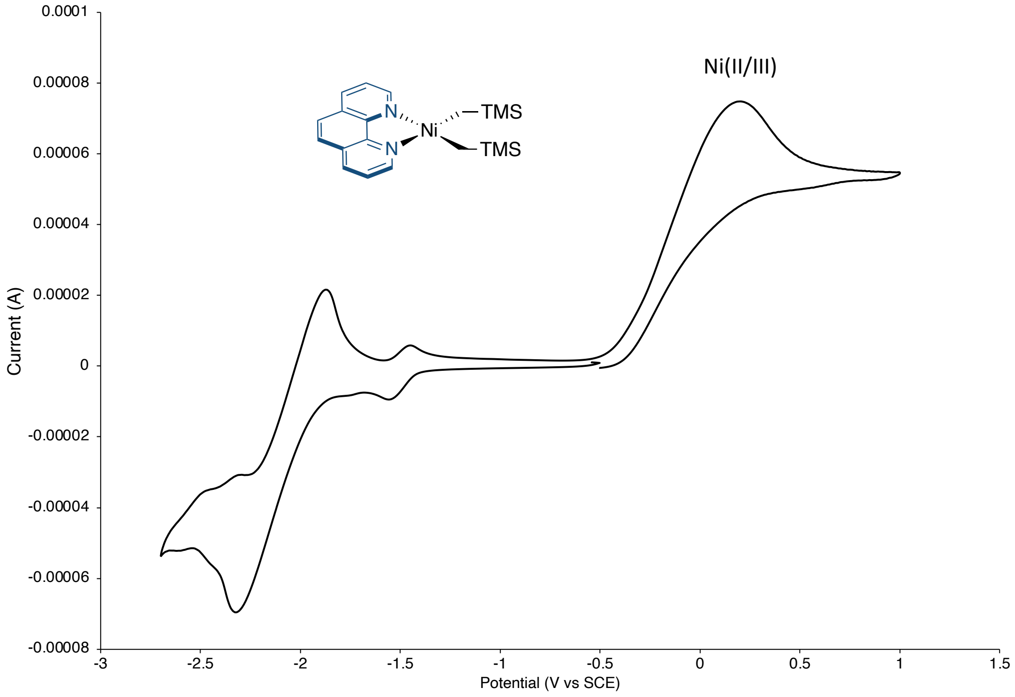

Figure S22. Cyclic voltammogram of (Phen)Ni(CH_2TMS)$_2$. Voltammograms were taken using a glassy carbon working electrode in a 0.1 M [$^nBu_4N$][PF_6] supporting electrolyte THF solution with a 100 mV/s scan rate and 0.01 M of sample referenced to Fc (+0.38 V vs SCE). Scans were started at the open-circuit potential and scanned in the anode direction first. E_p value for (Phen)Ni(CH_2TMS)$_2$ Ni(II)/Ni(III) = 0.22 V vs SCE.

Go to: [table of contents] [references]

S5. NMR Spectra of Synthesized Complexes

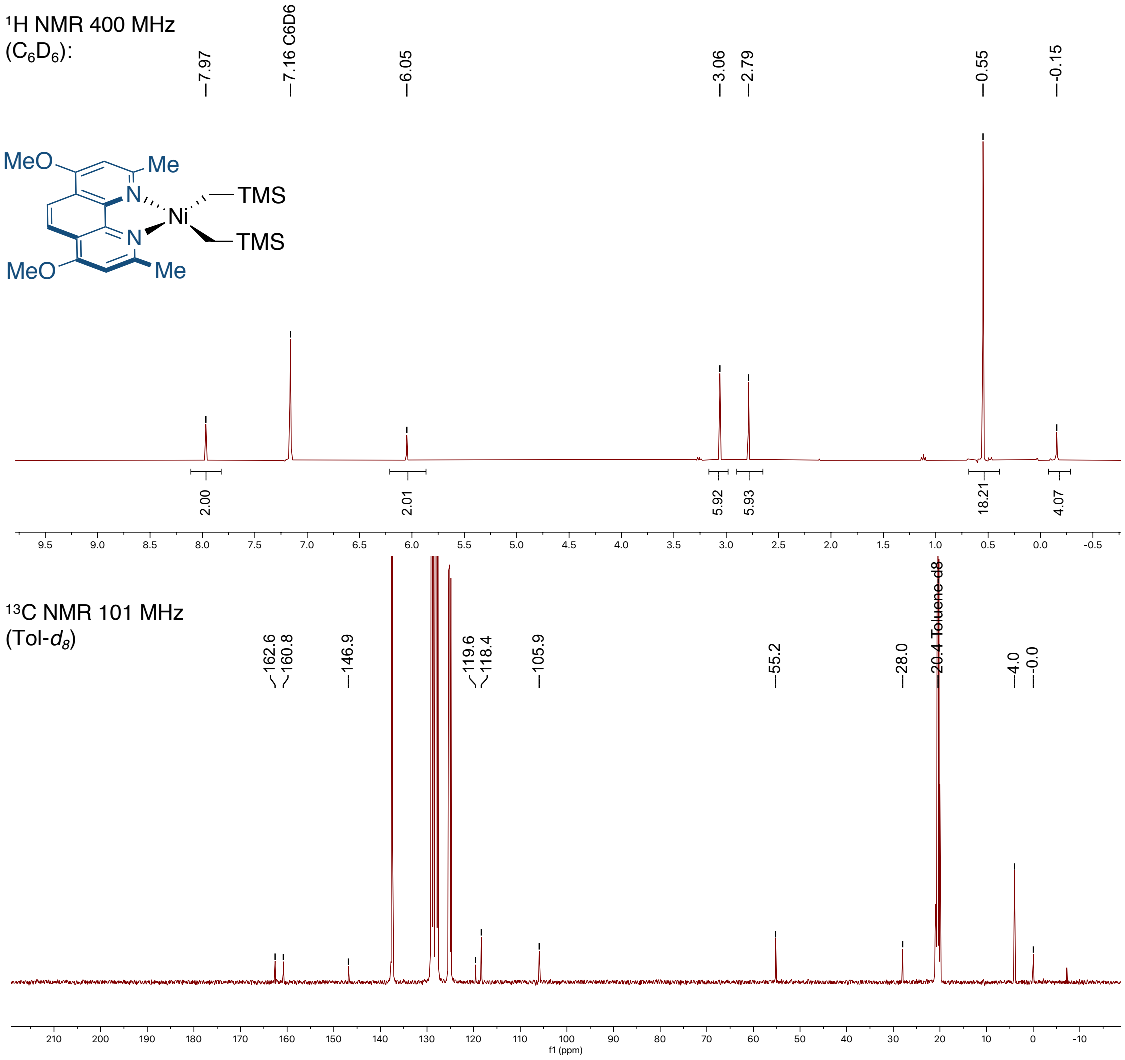

Figure S23. ^{1}H NMR (C_6D_6, 400 MHz) and ^{13}C NMR (Tol-d_8, 101 MHz) of (4,7-dimethoxy-2,9-dimethyl-1,10-phenanthroline)$Ni(CH_2TMS)_2$.

S6. Crystallographic Data

Data collection: The measured crystals were prepared under inert conditions immersed in perfluoropolyether as protecting oil for manipulation.

Crystal structure determination were carried out using a Rigaku diffractometer equipped with a Pilatus 200K area detector, a Rigaku MicroMax-007HF microfocus rotating anode with MoK_{α} radiation, Confocal Max Flux optics and an Oxford Cryosystems low temperature device Cryostream 700 plus (T = -173 °C). Full-sphere data collection was used with ω and φ scans. *Programs used:* Data collection data reduction with CrysAlisPro[4] and absorption correction with Scale3 Abspack scaling algorithm.[5]

Structure Solution and Refinement: Crystal structure solution was achieved using the computer program SHELXT.[6] Visualization was performed with the program SHELXle.[7] Missing atoms were subsequently located from difference Fourier synthesis and added to the atom list. Least-squares refinement on F^2 using all measured intensities was carried out using the program SHELXL 2015.[8] All non-hydrogen atoms were refined including anisotropic displacement parameters.

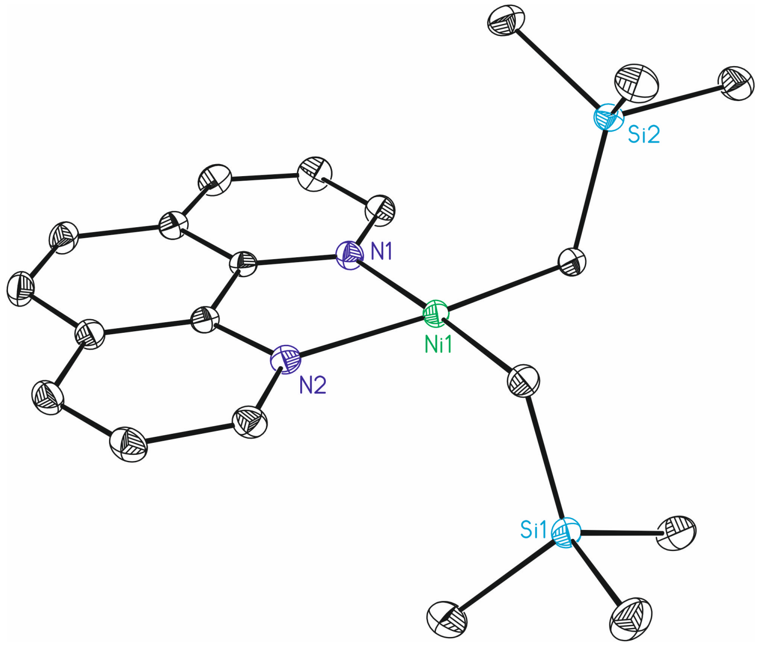

Figure S24. ORTEP drawing (50 %) showing (Phen)Ni(CH_2TMS_2)$_2$. Hydrogen atoms and disordered parts have been omitted in the sake of clarity. CCDC deposition number **CCDC-2191651.**

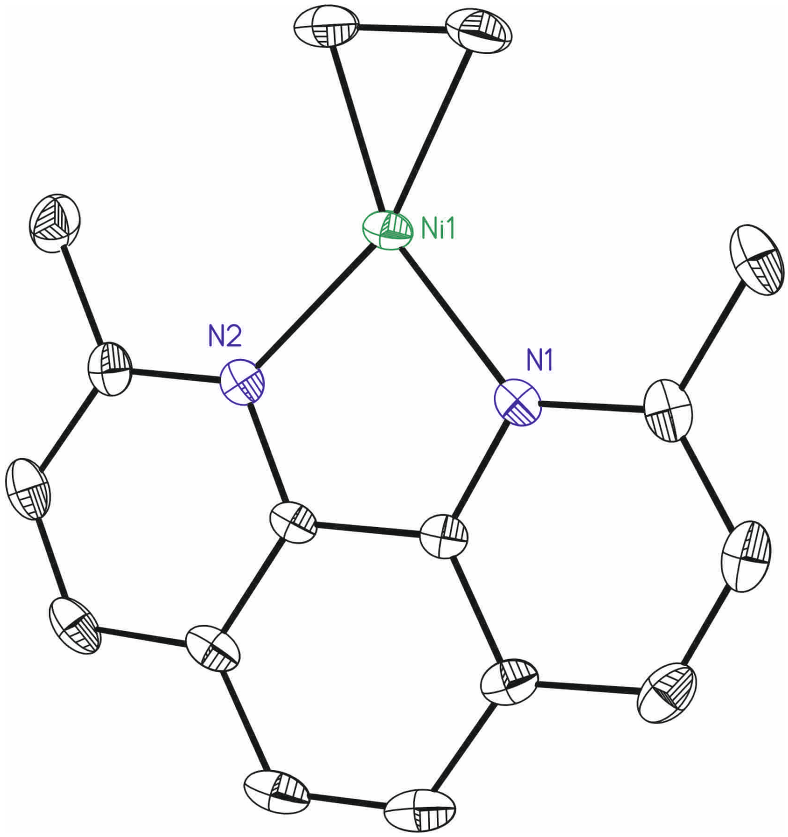

Figure S25. ORTEP drawing (50 %) showing (Neocuproine)Ni(C_2H_4). Hydrogen atoms and disordered parts have been omitted in the sake of clarity. CCDC deposition number **CCDC-2191653.**

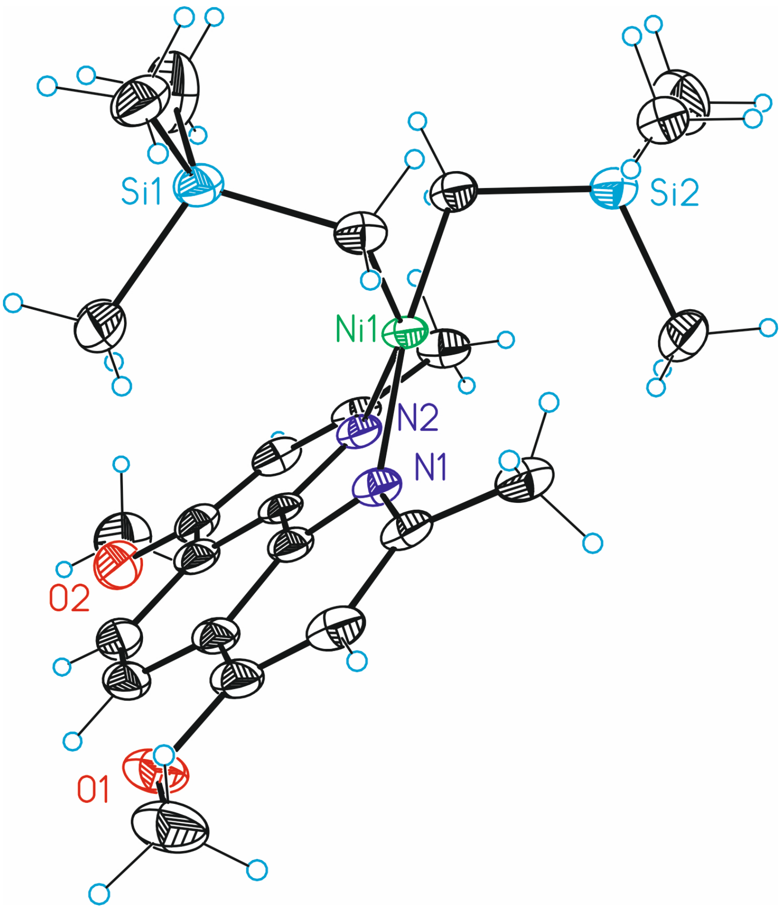

Figure S26. ORTEP drawing (50 %) showing (L4)Ni(CH_2TMS)$_2$. Disordered parts have been omitted in the sake of clarity. CCDC deposition number **CCDC-2191652.**

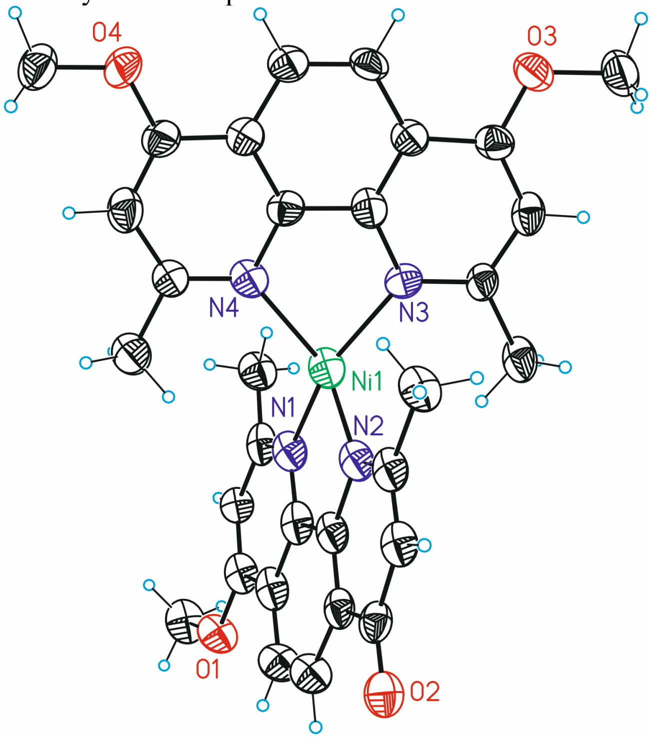

Figure S27. ORTEP drawing (50 %) showing (L4)(7-methoxy-2,9-dimethyl-1,10-phenanthrolin-4-ol)Ni. Solvent and disordered parts have been omitted in the sake of clarity. CCDC deposition number **CCDC-2191654.**

Table S1. Crystallographic Data

	**(Phen)Ni(CH$_2$TMS$_2$)$_2$**	**(Neocuproine)Ni(C$_2$H$_4$)**
**Formula**	$C_{20}H_{30}N_2NiSi_2$	$C_{16}H_{16}N_2Ni$
**Formula weight**	413.35	295.02
**T (K)**	100(2)	100(2)
**Wavelength (Å)**	0.71073	0.71073
**Crystal system**	Monoclinic	Orthorhombic
**Space group**	*P*21/n	*P*bca
a **(Å)**	7.16860(10)	18.0679(5)
b **(Å)**	12.0367(2)	7.4053(2)
c **(Å)**	24.5788(3)	19.1575(6)
**α (deg)**	90	90
**β (deg)**	94.8870(10)	90
**γ (deg)**	90	90
**V (Å^3)**	2113.10(5)	2563.24(13)
**Z**	4	8
**Density (calc.) (Mg/m^3)**	1.299	1.529
**μ (mm^{-1})**	1.037	1.499
**F(000)**	880	1232
**Crystal size (mm^3)**	0.100 x 0.100 x 0.100	0.250 x 0.200 x 0.100
**Theta range for data collection (deg)**	2.373 to 31.802	2.126 to 33.737
**Index ranges**	-10<=h<=10, -17<=k<=17, -34<=l<=36	-27<=h<=28, -8<=k<=11, -29<=l<=29
**Reflections collected**	41226	54015
**Independent reflections**	6893[R(int) = 0.0211]	5120[R(int) = 0.0280]
**Completeness to theta**	95.4 % 31.802°	100.0 % 33.737°
**Absorption correction**	Multi-scan	Multi-scan
**Max. and min. transmission**	1.00 and 0.37	0.74 and 0.63
**Refinement method**	Full-matrix least-squares on F^2	Full-matrix least-squares on F^2
**Data / restraints / parameters**	6893/ 0/ 232	5120/ 0/ 174
**Goodness-of-fit on F^2**	1.273	1.085
**Final R indices [I>2sigma(I)]**	R1 = 0.0372, wR2 = 0.0932	R1 = 0.0334, wR2 = 0.0915
**R indices (all data)**	R1 = 0.0389, wR2 = 0.0937	R1 = 0.0387, wR2 = 0.0951
**Largest diff. peak and hole**	0.748 and -0.500 e.Å^{-3}	1.116 and -0.367 e.Å^{-3}

Go to: [table of contents] [references]

Table S2. Crystallographic Data

	**(L4)(7-methoxy-2,9-dimethyl-1,10-phenanthrolin-4-ol)Ni**	**(L4)Ni(CH2TMS)$_2$**
**Formula**	$C_{35}H_{37}N_4NiO_5$	$C_{24}H_{38}N_2NiO_2Si_2$
**Formula weight**	652.39	501.45
**T (K)**	100(2)	100(2)
**Wavelength (Å)**	0.71073	0.71073
**Crystal system**	Triclinic	Monoclinic
**Space group**	*P*-1	C2/*c*
***a* (Å)**	8.930(4)	18.815(4)
***b* (Å)**	13.167(6)	11.930(3)
***c* (Å)**	13.700(6)	23.832(5)
**α (deg)**	110.938(11)	90
**β (deg)**	91.883(10)	92.970(7)
**γ (deg)**	91.427(12)	90
**V (Å^3)**	1502.6(11)	5342(2)
**Z**	2	8
**Density (calc.) (Mg/m^3)**	1.442	1.247
**μ (mm^{-1})**	0.697	0.838
**F(000)**	686	2144
**Crystal size (mm^3)**	0.200 x 0.030 x 0.010	0.200 x 0.100 x 0.040
**Theta range for data collection (deg)**	1.593 to 26.088	1.711 to 26.974
**Index ranges**	-10<=h<=11, -16<=k<=16, -16<=l<=16	-23<=h<=24, -15<=k<=15, -30<=l<=30
**Reflections collected**	10684	63021
**Independent reflections**	5756[R(int) = 0.0974]	5759[R(int) = 0.1255]
**Completeness to theta**	96.6 % 26.088 °	99.0 % 26.974 °
**Absorption correction**	Multi-scan	Multi-scan
**Max. and min. transmission**	0.74 and 0.44	0.74 and 0.55
**Refinement method**	Full-matrix least-squares on F^2	Full-matrix least-squares on F^2
**Data / restraints / parameters**	5756/ 6/ 413	5759/ 120/ 346
**Goodness-of-fit on F^2**	1.013	1.045
**Final R indices [I>2sigma(I)]**	R1 = 0.1148, wR2 = 0.2624	R1 = 0.0510, wR2 = 0.1152
**R indices (all data)**	R1 = 0.2057, wR2 = 0.3176	R1 = 0.0866, wR2 = 0.1350
**Largest diff. peak and hole**	2.397 and -0.593 e.Å^{-3}	0.883 and -0.608 e.Å^{-3}

S7. Computational details

The molecular structures were optimized using B3LYP[9-10] density functional combined with def2-TZVPP[11-12] basis set. The nature of local minima was confirmed by checking the Hessian matrix of energy. All DFT computations were performed by Gaussian 16 Rev. C1 suite of programs.[13] Chemcraft software is used for visualization of molecular orbitals. The wavefunctions of the systems were analyzed within the context of the quantum theory of atoms in molecules (QTAIM)[14] by AIMAll package.[15]

S8. References

1. Carmona, E.; González, F.; Poveda, M. L.; Atwood, J. L.; Rogers, R. D. Synthesis and properties of dialkyl complexes of nickel(II). The crystal structure of bis(pyridine)bis(trimethylsilylmethyl)nickel(II). *J. Chem. Soc., Dalton Trans.* **1981,** (3), 777-782.
2. Powers, D. C.; Anderson, B. L.; Nocera, D. G. Two-Electron HCl to H_2 Photocycle Promoted by Ni(II) Polypyridyl Halide Complexes. *J. Am. Chem. Soc.* **2013,** *135* (50), 18876-18883.
3. Liu, X.; Li, X.; Chen, Y.; Hu, Y.; Kishi, Y. On Ni Catalysts for Catalytic, Asymmetric Ni/Cr-Mediated Coupling Reactions. *J. Am. Chem. Soc.* **2012,** *134* (14), 6136-6139.
4. Data reduction with CrysAlisPro 1.171.40.35 (Rigaku OD, 2018).
5. Empirical absorption correction using spherical harmonics implemented in Scale3 Abspack scaling algorithm, CrysAlisPro 1.171.40.35 (Rigaku OD, 2018).
6. Sheldrick, G. M. SHELXT– Integrated space-group and crystal-structure determination. *Acta Crystallographica Section A Foundations and Advances* **2015,** *71* (1), 3-8.
7. Hübschle, C. B.; Sheldrick, G. M.; Dittrich, B. ShelXle: a Qt graphical user interface forSHELXL. *J. Appl. Crystallogr.* **2011,** *44* (6), 1281-1284.
8. Sheldrick, G. M. Crystal structure refinement withSHELXL. *Acta Crystallographica Section C Structural Chemistry* **2015,** *71* (1), 3-8.
9. Becke, A. D. Density-functional thermochemistry. III. The role of exact exchange. *J. Chem. Phys.* **1993,** *98* (7), 5648-5652.
10. Lee, C.; Yang, W.; Parr, R. G. Development of the Colle-Salvetti correlation-energy formula into a functional of the electron density. *Physical Review B* **1988,** *37* (2), 785-789.
11. Weigend, F.; Ahlrichs, R. Balanced basis sets of split valence, triple zeta valence and quadruple zeta valence quality for H to Rn: Design and assessment of accuracy. *Physical Chemistry Chemical Physics* **2005,** *7* (18), 3297-3305.
12. Weigend, F. Accurate Coulomb-fitting basis sets for H to Rn. *Physical Chemistry Chemical Physics* **2006,** *8* (9), 1057-1065.
13. Frisch, M. J.; Trucks, G. W.; Schlegel, H. B.; Scuseria, G. E.; Robb, M. A.; Cheeseman, J. R.; Scalmani, G.; Barone, V.; Petersson, G. A.; Nakatsuji, H.; Li, X.; Caricato, M.; Marenich, A. V.; Bloino, J.; Janesko, B. G.; Gomperts, R.; Mennucci, B.; Hratchian, H. P.; Ortiz, J. V.; Izmaylov, A. F.; Sonnenberg, J. L.; Williams; Ding, F.; Lipparini, F.; Egidi, F.; Goings, J.; Peng, B.; Petrone, A.; Henderson, T.; Ranasinghe, D.; Zakrzewski, V. G.; Gao, J.; Rega, N.; Zheng, G.; Liang, W.; Hada, M.; Ehara, M.; Toyota, K.; Fukuda, R.; Hasegawa, J.; Ishida, M.; Nakajima, T.; Honda, Y.; Kitao, O.; Nakai, H.; Vreven, T.; Throssell, K.; Montgomery Jr., J. A.; Peralta, J. E.; Ogliaro, F.; Bearpark, M. J.; Heyd, J. J.; Brothers, E. N.; Kudin, K. N.; Staroverov, V. N.; Keith, T. A.; Kobayashi, R.; Normand, J.; Raghavachari, K.; Rendell, A. P.; Burant, J. C.; Iyengar, S. S.; Tomasi, J.; Cossi, M.; Millam, J. M.; Klene, M.; Adamo, C.; Cammi, R.; Ochterski, J. W.; Martin, R. L.; Morokuma, K.; Farkas, O.; Foresman, J. B.; Fox, D. J. *Gaussian 16 Rev. C.01*, Wallingford, CT, 2016.
14. Bader, R. F. W., *Atoms in Molecules: A Quantum Theory.* Clarendon Press: Oxford, 1990.
15. Keith, T. A. *AIMAll*, Version 19.10.12; TK Gristmill Software: Overland Park KS, USA, 2019.
